# Evaluation of ATM Kinase Inhibitor KU-55933 as Potential Anti-*Toxoplasma gondii* Agent

**DOI:** 10.3389/fcimb.2019.00026

**Published:** 2019-02-13

**Authors:** Jonathan Munera López, Agustina Ganuza, Silvina S. Bogado, Daniela Muñoz, Diego M. Ruiz, William J. Sullivan, Laura Vanagas, Sergio O. Angel

**Affiliations:** ^1^Laboratorio de Parasitología Molecular, IIB-INTECH, Consejo Nacional de Investigaciones Científicas (CONICET)-Universidad Nacional General San Martin (UNSAM), Chascomús, Argentina; ^2^Pharmacology and Toxicology, Indiana University School of Medicine, Indianapolis, IN, United States; ^3^Microbiology and Immunology, Indiana University School of Medicine, Indianapolis, IN, United States

**Keywords:** *Toxoplasma gondii*, DNA repair, cell cycle, fork collapse, antiparasitic drugs

## Abstract

*Toxoplasma gondii* is an apicomplexan protozoan parasite with a complex life cycle composed of multiple stages that infect mammals and birds. Tachyzoites rapidly replicate within host cells to produce acute infection during which the parasite disseminates to tissues and organs. Highly replicative cells are subject to Double Strand Breaks (DSBs) by replication fork collapse and ATM, a member of the phosphatidylinositol 3-kinase (PI3K) family, is a key factor that initiates DNA repair and activates cell cycle checkpoints. Here we demonstrate that the treatment of intracellular tachyzoites with the PI3K inhibitor caffeine or ATM kinase-inhibitor KU-55933 affects parasite replication rate in a dose-dependent manner. KU-55933 affects intracellular tachyzoite growth and induces G1-phase arrest. Addition of KU-55933 to extracellular tachyzoites also leads to a significant reduction of tachyzoite replication upon infection of host cells. ATM kinase phosphorylates H2A.X (γH2AX) to promote DSB damage repair. The level of γH2AX increases in tachyzoites treated with camptothecin (CPT), a drug that generates fork collapse, but this increase was not observed when co-administered with KU-55933. These findings support that KU-55933 is affecting the *Toxoplasma* ATM-like kinase (TgATM). The combination of KU-55933 and other DNA damaging agents such as methyl methane sulfonate (MMS) and CPT produce a synergic effect, suggesting that TgATM kinase inhibition sensitizes the parasite to damaged DNA. By contrast, hydroxyurea (HU) did not further inhibit tachyzoite replication when combined with KU-55933.

## Introduction

*Toxoplasma gondii* is a widespread protozoan parasite that infects humans and warm-blooded animals. Although the course of toxoplasmic infection is usually asymptomatic, severe problems, and even death can occur in immunocompromised individuals (e.g., AIDS, transplantation) or as a result of congenital infection. In HIV patients, reactivation of the infection can cause neurological defects, encephalitis, and chorioretinitis; congenital toxoplasmosis is responsible for neurological defects, chorioretinitis, and in some cases abortion (Luft and Remington, 1992; Moncada and Montoya, 2012). The life cycle of *Toxoplasma* includes the sexual stage (sporozoite), which occurs only in the definitive host (felines), and asexual stages (tachyzoite and bradyzoite), both occurring in all mammals and birds (Dubey, 1994). It is generally accepted that the highly replicative tachyzoites produce clinical symptoms whereas the bradyzoites (which reside within intracellular tissue cysts) cause the asymptomatic latent infection with the ability to reconvert into tachyzoites. However, recent associations have been made between chronic *Toxoplasma* infection and neurological disorders, such as schizophrenia (Torrey et al., 2012; Sutterland et al., 2015; Flegr and Horacek, 2017; Fuglewicz et al., 2017; Yolken et al., 2017).

The frontline treatment for toxoplasmosis includes anti-folate drugs, which are only effective against the tachyzoite stage and produce serious adverse effects and allergic reactions (Luft and Remington, 1992; Carlier et al., 2012). There is no effective treatment for chronic toxoplasmosis as no drug is known to eliminate tissue cysts. Newer, safer drugs effective in treating toxoplasmosis are urgently needed.

Rapidly replicating cells such as tachyzoites must contend with DNA damage. *Toxoplasma* tachyzoites cultured *in vitro* show detectable basal levels of γH2A.X, a marker of DNA damage, as revealed by Western blot and mass spectrometry analysis (Dalmasso et al., 2009; Nardelli et al., 2013). Histone H2AX is a H2A variant with a SQE C-terminal motif that can be modified by a kinase, generating the phosphorylated form γH2A.X. The spreading of γH2A.X at both sides of a double strand break (DSB) is one of the earliest events involved in the DNA damage response (DDR) to different genotoxic stresses and occupies megabase chromatin domains (Rogakou et al., 1998, 1999; Redon et al., 2002; Martin et al., 2003). H2A.X phosphorylation is mediated by members of phosphatidyl-inositol 3-kinase family (PI3K) such as Ataxia telangiectasia mutated (ATM) kinase, ATM Rad-3-related (ATR), and DNA dependent protein kinase (DNA-PK). ATM kinase and DNA-PK are involved mainly in DSB repair whereas ATR is associated with single strand DNA (ssDNA) and stalled replication forks (Branzei and Foiani, 2008). ATM is the key kinase for H2A.X phosphorylation after DSB, and also phosphorylates other cell cycle and DDR proteins, allowing the γH2A.X foci generation and DDR either by non-homologous end joining (NHEJ) or homologous recombination repair (HRR) (Bakkenist and Kastan, 2003). DNA-PK is activated through its interaction with Ku and is associated with the NHEJ pathway (Pannunzio et al., 2017), however, DNA-PK and ATM kinase have overlapping functions to phosphorylate H2A.X after ionizing radiation DNA damage (Stiff et al., 2004; Wang et al., 2005). ATM kinase also phosphorylates H2A.X and DNA-PK in response to DSB produced by the topoisomerase I inhibitor camptothecin (CPT) or topoisomerase II inhibitor mitoxantrone (Kurose et al., 2005; Cristini et al., 2016). Various cellular mechanisms work to ensure the integrity of the genome during DNA replication, but sometimes fork stalling occurs and generates ssDNA. In the event that the lesion cannot be repaired, the forks collapse, generating one-end DSB that requires DDR. Among factors that are recruited to one-end DSB are the Mre11-Rad50-Nbs1/Xrs2 complex and ATM kinase (Lee and Paull, 2005). DSBs produced by fork collapse generated by topoisomerase I inhibitor topotecan require ATM kinase for the completion of HRR (Kurose et al., 2005; Tanaka et al., 2006; Kocher et al., 2013). γH2A.X can also appear by chemical and environmental agents that do not induce DSBs, such as benz[a]pyrene, which leads to formation of covalent DNA adducts. In this case, H2A.X phosphorylation has shown to be induced by ATM, ATR, or DNA-PK kinases (Yan et al., 2011). Hyperthermia and heat shock can also cause ATM-dependent γH2A.X induction (Hunt et al., 2007; Takahashi et al., 2010). Among targets of ATM kinase is Hsp90a; phosphorylation of Hsp90a at threonine 5 and 7 correlates with an increase in γH2A.X (Elaimy et al., 2016).

The *Toxoplasma* ATM (TgATM) kinase (Vonlaufen et al., 2010) seems to be essential as observed by a CRISPR-screen assay (Sidik et al., 2016), along with other PI3Ks (Table 1). These findings suggest an important biological role for such kinases under normal growth conditions. There are several compounds (caffeine, KU-55933 and derivatives) that have shown inhibitory effects against PI3K kinases and were studied as promising candidates for cancer therapy (Bode and Dong, 2007; Kuroda et al., 2012; Batey et al., 2013; Teng et al., 2015). Caffeine is a non-specific PI3K inhibitor whose targets include ATM kinase at IC_50_ of 0.2 mM, ATR kinase at IC_50_ of 1.1 mM, DNA-PK at IC_50_ between 0.2 and 0.6 mM (Block et al., 2004), and other targets (Bode and Dong, 2007). By contrast, KU-55933 is a potent and selective ATP-competitor of ATM kinase at IC_50_ of 12.9 nM (Hickson et al., 2004).

**Table 1:** Domain structure of *T. gondii* phosphatidylinositol 3- and 4-kinase (PIKK) domain-containing proteins.

**Gene ID (e.g., TGME49)**	**NLS**	**FAT**	**PRD**	**FATC**	**MW (kDa)**	**Blastp**	**Phenotype Score^a^**
_248530	2	NO	ND	1	246	HuATM	−2.71
_266010	1	NO	ND	1	964	DNA-PK	−3.15
_268370	2	1	1	1	904	HuTRRAP	−3.56
_283702	NO	NO	1	1	647	HuATR	−2.68
_316430	2	1	1	1	543	mTOR	0.21

*These PI3,4K putative proteins were retrieved from www.toxodb.org based on Blast analysis by using Human ATM (AAB65827), ATR (CAA70298), and DNA-PK (AAB39925) aminoacidic sequences. Domains and motifs were searched by using motifscan (http://hits.isb-sib.ch/cgi-bin/PFSCAN). FAT, FRAP (FKBP12-rapamycin-associated protein)-ATM-TRRAP (Transformation/transcription domain-associated protein) domain; PRD, PIKK regulatory domain; FATC, FAT-C-terminal domain. HuTRRAP, transformation/transcription domain-associated protein, isoform CRA_e (EAW76697). mTOR: mammalian Target Of Rapamycin (NP_004949). mRNA sequence is obtained by exon/intron prediction on genome sequence. For this reason, the gene, ORF, and protein sequences are subjected to future modifications*.

ND: not detected

a *“Genome-wide loss of function screen (CRISPR) that measures each gene's contribution to Toxoplasma gondii fitness during infection of human fibroblasts. Phenotype score = log2 (sgRNA of infected cultures/sgRNA composition of original library)” (www.toxodb.org). Negative score, fitness conferring; positive score, dispensable*.

DNA replication and repair pathways are promising drug targets for the development of novel antiparasitic. In the present study, we analyzed the effect of the ATM kinase inhibitors caffeine and KU-55399 on tachyzoites *in vitro*. We observed that both inhibitors impair *T. gondii* replication. The presence of KU-55933 also inhibits H2A.X phosphorylation in intracellular tachyzoites cultured in presence of camptothecin (CPT), a topoisomerase I venom (Hickson et al., 2004; Tomicic and Kaina, 2013; Botella and Rivero-Buceta, 2017). The combination of KU-55933 and DNA damaging agents such as CPT or methyl methane sulfonate (MMS) showed a synergic effect in slowing parasite growth. The impact of our findings in light of the discovery of future drug targets in toxoplasmosis is discussed.

## Materials and Methods

### Parasite Culture

Wild-type RH strain parasites and RH RFP, which express red fluorescent protein (van Dooren et al., 2008), were cultured in standard tachyzoite conditions *in vitro*: human foreskin fibroblast (HFF) monolayers were infected with tachyzoites and incubated in Dulbecco's modified Eagle medium (DMEM, GIBCO) supplemented with 10% fetal bovine serum, penicillin (100 UI/ml; GIBCO), and streptomycin (100 μg/ml; GIBCO) at 37°C and 5% CO_2_.

### Chemicals and Antibodies

Camptothecin (CPT, Sigma-Aldrich Argentina, catalog number C9911) was dissolved in DMSO at a concentration of 1 mM and stored at −20°C as stock solution. Caffeine (Sigma-Aldrich Argentina, catalog number C0750) was dissolved in water at a concentration of 100 mM and stored at −20°C as stock solution. KU-55933 (Calbiochem catalog number 118500) was dissolved in DMSO at a concentration of 10 mM and stored at −20°C as stock solution. Hydroxyurea (HU, Sigma-Aldrich Argentina, catalog number H8627-5G) was dissolved in water at a concentration of 50 mg/ml as stock solution and disposed after use. Methyl methane sulfonate (MMS, Sigma-Aldrich Argentina, catalog number 129925-5G, liquid, 11,8 M) was dissolved in DMEM at the concentrations indicated for each assay and disposed after use.

Anti-γH2AX antibody was obtained from Merck Argentina (JBW301). Rabbit anti-*Toxoplasma* H2A.X and Hsp90 were previously produced in our laboratory (Echeverria et al., 2005; Dalmasso et al., 2009). Anti-actin antibody was kindly provided by Jean F. Dubremetz (Université de Montpellier, Montpellier, France). Murine anti-SAG1 antibody was kindly provided by Marina Clemente (Albarracín et al., 2015). Mouse monoclonal anti-H3 antibody was purchased from Abcam (10799). Alexa fluor goat antibodies anti-mouse 594 (A-11032), anti-rabbit 594 (A-11037), anti-mouse 488 (A-11001), and anti-rabbit 488 (A-11034) were purchased from Invitrogen.

### Replication Assay

The replication rate was determined in infected monolayers, treated or untreated with different doses of caffeine, KU-55933 or CPT. Coverslips with confluent HFFs were infected with 1 × 10^4^ parasites (MOI: 0.1 Tachyzoites/host cell). After 1 h of incubation, cells were washed three times with PBS and incubated 12–48 h in DMEM plus treatment, then cells were analyzed by indirect immunofluorescence (IFA) to facilitate counting. Briefly, they were fixed with 4% (v/v) paraformaldehyde and blocked with 1% BSA. Primary antibodies anti-SAG1 diluted 1:100 with 0.5% BSA or anti-*T. gondii* Hsp90 1:2,000 were incubated at room temperature for 1 h. After several washes with PBS, they were incubated with secondary antibodies Alexa fluor goat anti-mouse 594 or Alexa fluor goat anti-mouse 488 (Invitrogen). Cover slips were washed three times and mounted in Fluoromont G (Southern Biotechnology Associates) and viewed using a Nikon Model Eclipse E600 (magnification 100X, numerical aperture 1,40 at 24°C). Green or red fluorescence were recorded separately and the images were analyzed by Image-Pro Plus version 5.1.0.20 and merged using Adobe Photoshop. Parasites in 100 randomly chosen parasitophorous vacuoles (PV) were counted in triplicate. Data are presented as the average number of tachyzoites *per* PV. IC_50_ was obtained by GraphPad Prism 6: data were normalized with 0 as the smallest value and transformed to semi-logarithmic scale [x = log(x)]. After that, they were analyzed as a nonlinear regression parameter-Dose-response inhibition-log(inhibitor) vs. normalized response-variable slope.

### RH RFP Fluorescence Assay

Fluorescence assay was carried out using an RH strain parasite clone engineered to express Red Fluorescent Protein (RFP), kindly provided by Silvia Moreno (University of Georgia, Athens, Georgia). RH RFP tachyzoites were used to infect HFF monolayer in a 96-well plate with or without the indicated drugs. Fluorescence values were measured 4 days post-infection and both excitation (544 nm) and emission (590 nm) were read from the bottom of the plates in a microplate reader (Synergy H1). Data were plotted and analyzed using GraphPad Prism 6 software.

### Cell Cycle

HFF cells were grown to confluence in 6 well plates then infected with 1 × 10^6^ RH tachyzoites per well and treated with 60 μM pyrrolidine dithiocarbamate (PDTS) for 6 h in DMEM (Conde de Felipe et al., 2008). Plates were then washed with PBS and incubated with 5 μM KU-55933, 4 mM HU, or 0.1%v/v DMSO for 7 h. Plates were washed with PBS and the cells were harvested with trypsin, passed through different sizes of needles to lyse the host cells and finally the parasites were filtered using a 3 μm filter. Purified parasites were centrifuged at 2,000 RPM for 10 min and washed with PBS, then fixed with 70% ethanol, and incubated 24 h at −20°C. Afterwards, samples were centrifuged and washed with PBS supplemented at 2% with FBS. After centrifugation again, they were resuspended in 1 ml of supplemented PBS + 180 μg / ml RNAse and incubated for 10 min at 37°C. Finally, they were incubated with Propidium Iodide (0.5mg / ml) for 10 min before carrying out the measurement in the BD FACS Calibur flow cytometer and analyzed by FlowJo 7.6.

### Immunoblotting

Proteins from purified parasites were resolved by SDS-PAGE and transferred onto a nitrocellulose membrane. Non-specific binding sites were blocked with 5% non-fat-dried milk in PBS containing 0.05% Tween-20 (PBS-T) and the membranes were then incubated (1 h at room temperature) with primary antibodies. The antibodies and dilutions used in this study were: murine anti-γH2AX (1:1000) from Millipore (05–636), anti-actin (1:500), anti-H3 (1:1,000), and antibodies produced by our laboratory: rabbit anti-H2A.X (1:5,000) (Dalmasso et al., 2009). The membranes were washed several times with TBS-T prior to incubation with alkaline phosphatase–conjugated anti-rabbit or anti-mouse secondary antibodies, diluted 1:10,000 (Santa Cruz Biotechnology). Immunoreactive protein bands were visualized by the NBT-BCIP method (Sigma-AldrichTM Argentina S.A). Intensities of bands were quantified from scanned images using ImageJ software.

## Results

### Effect of Caffeine PI3K Inhibitor on Tachyzoite Replication and Growth

**Figure 1:**
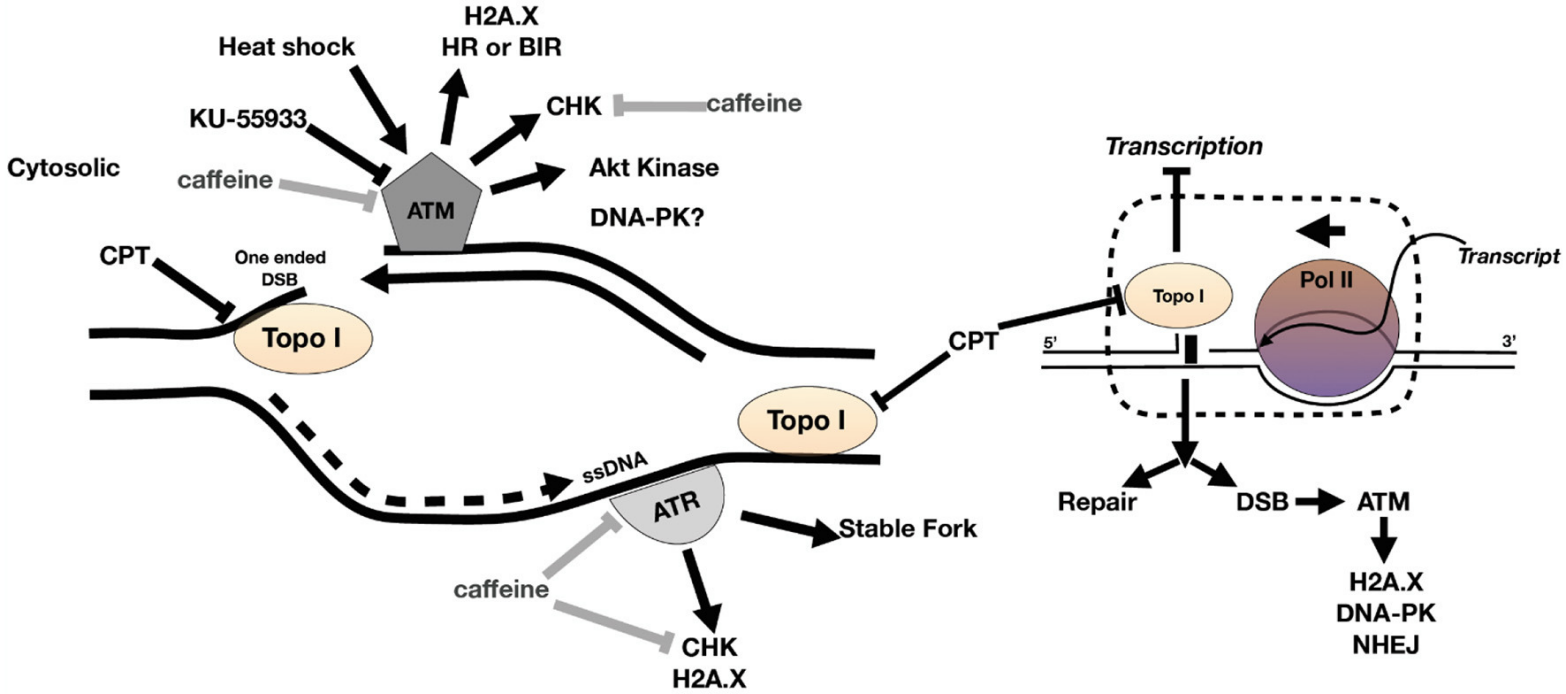
Model of DNA damage at fork during replication. ATR binds to ssDNA at a stalled fork to stabilize the fork. ATM kinase binds to one-ended DSB at collapsed fork. DNA damage activates ATM and ATR to phosphorylate DNA damage response (DDR) proteins such as H2A.X and checkpoint kinases, the latter blocking the cell cycle until DNA is repaired (or apoptosis commences). The collision of replication fork and transcription fork can also generate DSB and recruitment of DDR factors including ATM kinase. CPT is a topoisomerase I (topo I) venom and can cause fork collapse and DSB during DNA replication. Caffeine inhibits ATM and ATR kinase activity and KU-55933 inhibits ATM kinase.

**Figure 2:**
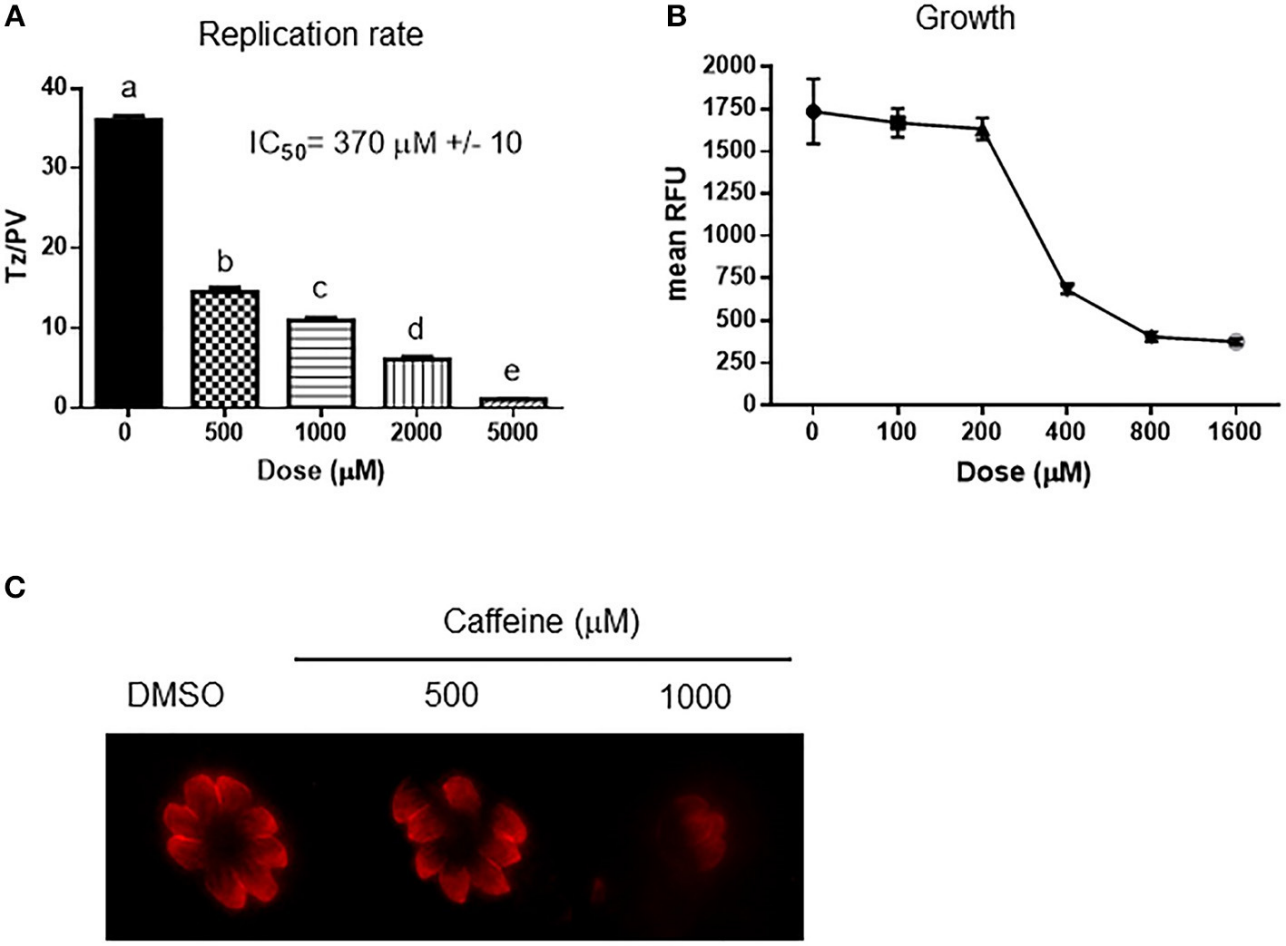
Effect of caffeine on *Toxoplasma* replication and growth. **(A)** Intracellular tachyzoites were grown in culture with DMSO or different doses of caffeine during 48 h. After that, they were fixed and stained with anti-tubulin. Tachyzoites *per* parasitophorous vacuole (Tz/PV) were counted in 100 randomly chosen vacuoles. Statistical analysis was performed by one-way ANOVA and Tukey's Multiple Comparison Test. Results are the mean of three replicates plus SD. Different letters indicate statistically significant differences between columns (*p* ≤ 0.05), according to one-way ANOVA, and Tukey's multiple comparison test. Details: *p* ≤ 0.001: a vs. b, c, d, and e; *p* ≤ 0.001: b vs. c, d, and e; *p* ≤ 0.001: c vs. d and e; *p* ≤ 0.001: d vs. e. The graph is representative of three independent experiments with similar results. **(B)** Intracellular tachyzoites from RH RFP strain were treated with Caffeine at different doses during 96 h and their growth analyzed at 544 nM. Results were plotted by GraphPad Prism 6. Results are mean of three replicates plus SD. **(C)** Arrangement of tachyzoites inside PV is visualized at different doses of caffeine. In presence of DMSO, or caffeine up to 1,000 μM the typical rosette organization could be observed. PV with similar number of tachyzoites were selected to compare.

The inhibition of PI3K kinases such as ATM and ATR can block the correct DDR at DSB (Figure 1). There is evidence of putative homologs of ATM, ATR, and DNA-PK PI3K kinases in *Toxoplasma* [Table 1 and (Vonlaufen et al., 2010)]. Based on human ATM domain organization (Stracker et al., 2013), the most similar regions among ATM/Tel1 kinases involve the PI3K domain (Figure S1). In order to test whether PI3K inhibitors affect tachyzoite replication, infected monolayers were treated with different doses of caffeine, which is a broad-spectrum kinase inhibitor with known activity against ATM, and ATR kinases (Sarkaria, 2003; Bode and Dong, 2007). Intracellular tachyzoites were incubated with caffeine for 48 h and then the number of parasites *per* parasitophorous vacuole (PV) was counted. Caffeine significantly slowed the tachyzoite replication rate in a dose-dependent manner with an IC_50_ = 370 μM (Figure 2A). In addition, the effect of caffeine on tachyzoite growth was also determined. Figure 2B shows that doses higher than 200 μM significantly affect tachyzoite growth and completely abolished it at 800 μM. MTT (3-(4,5-dimethylthiazol-2-yl)-2,5-diphenyl-tetrazolium bromide) assay did not evidence impact of caffeine on HFF metabolism (Figure S2). In addition, caffeine did not disturb neither shape nor “rosette” organization of tachyzoites within PV (Figure 2C).

### Effect of ATM Kinase Inhibitor KU-55933 in *Toxoplasma* Cell Cycle and Growth

**Figure 3:**
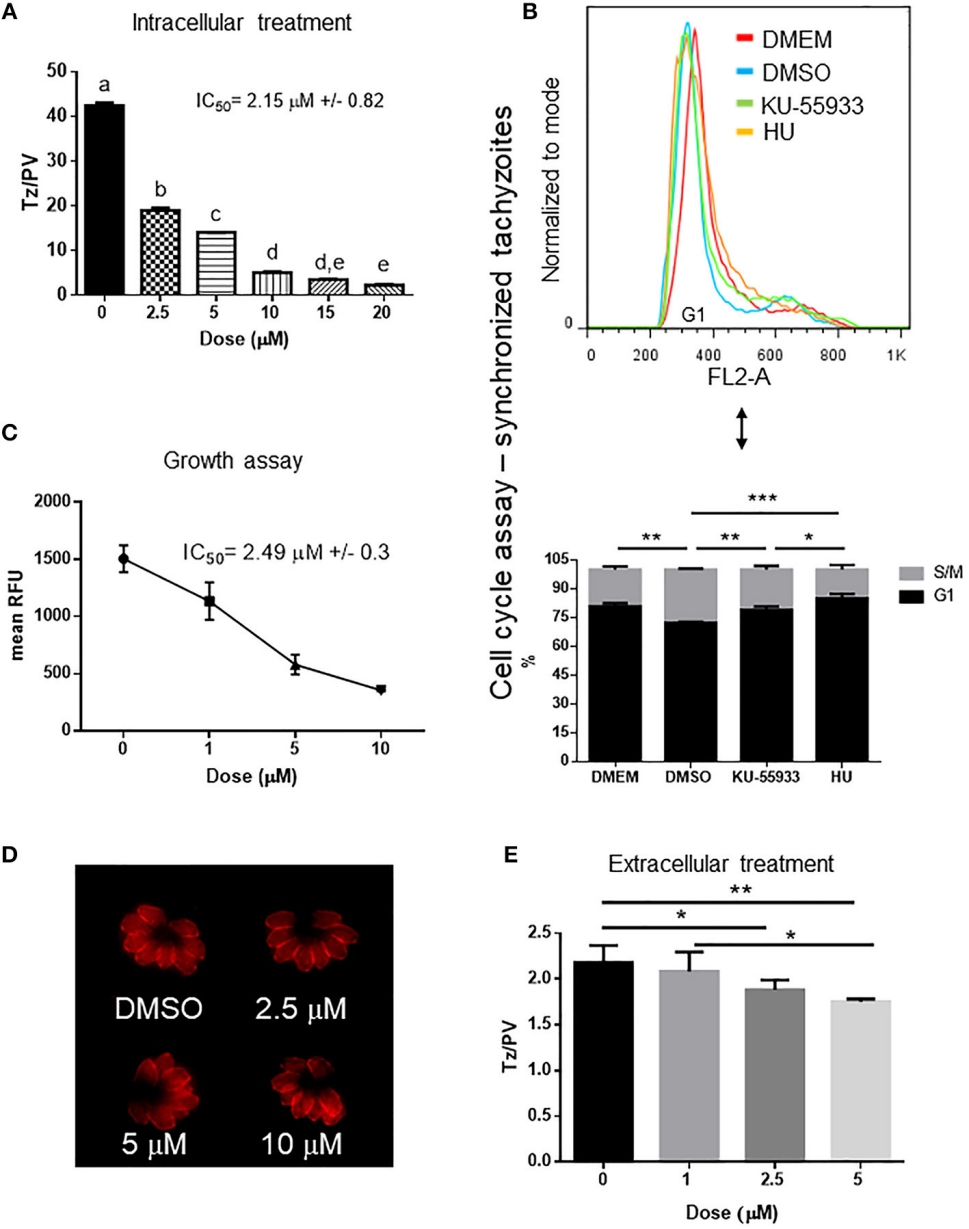
Effect of ATM kinase inhibitor KU-55933 on *Toxoplasma* replication and growth. Intracellular tachyzoites were grown in culture with DMSO or different doses of KU-55933 during 48 h. After that, they were fixed and stained with anti-tubulin. **(A)** Tachyzoites per PV (Tz/PV) were counted in 100 PV. Statistical analysis was performed by one-way ANOVA and Tukey's Multiple Comparison Test. Results are mean of three replicates plus SD. Same letters above the column indicate no significant differences; different letters indicate statistically significant differences between columns (*p* ≤ 0.05), according to one-way ANOVA, and Tukey's multiple comparison test. Details: *p* ≤ 0.0001: a vs. b, c, d, and e; *p* ≤ 0.0001: b vs. c, d, and e; *p* ≤ 0.0001: c vs. d and e; *p* ≤ 0.01: d vs. e. The graph is representative of three independent experiments with similar results. **(B)** Tachyzoites were added to confluent HFF host cells during 16 h and treated with PDTS for 6 h. Plates were then washed with PBS and incubated with 5 μM KU-55933, 4 mM HU or 0.1%v/v DMSO for 7 h and propidium iodide used to stain DNA. The tachyzoites were analyzed by FACS and DNA content was determined (G1: 1N). Statistical analysis was performed with one-way ANOVA and Tukey's multiple comparison test (*p ≤ 0.05; **p ≤ 0.01, and ***p ≤ 0.001). **(C)** Intracellular tachyzoites (RH RFP strain) were cultured in presence of DMSO or different doses of μM KU-55933 in 96-well plates for 4 days and then read at 544 nm (bottom of the plate) in a microplate reader. Results are means of six replicates plus SD. The graph is representative of three independent experiments with similar results. **(D)** Arrangement of tachyzoites inside PV is visualized at different doses of KU-55933. In presence of DMSO or the drug the typical rosette organization could be observed. PV with similar number of tachyzoites were selected to compare. **(E)** Extracellular tachyzoites were incubated for 4 h with DMSO or different doses of KU-55933. After that, they were added to HFF monolayers and incubated for 12 h in normal conditions. Replication rate was analyzed as in **(A)**. Statistical analysis was performed with one-way ANOVA and Tukey's multiple comparison test (*p ≤ 0.05; **p ≤ 0.01). Results are mean of three replicates plus SD.

As caffeine likely has multiple PI3K targets that could adversely affect parasite replication, we sought to test whether KU-55933, an established and selective ATM kinase inhibitor, had an effect on tachyzoite growth *in vitro*. Our findings show that *Toxoplasma* replication was affected by KU-55933 in a dose dependent manner with an IC_50_ = 2.15 μM (Figure 3A).

In order to study how KU-55933 affects the tachyzoite cell cycle, intracellular parasites were grown in presence of PDTS to synchronize the tahcyzoites in G1. After releasing of PDTS treatment intracellular tachyzoites were grown for 7 h in the presence of DMSO, 5 μM KU-55933, or 4 mM HU. Following treatment with KU-55933, parasites show a significant enrichment in DNA content compatible with G1-phase in comparison with the observed in the control and similar to the observed with HU and tachyzoites arrested in G1 (DMEM group) (Figure 3B).

To confirm the effect observed in tachyzoites treated with KU-55933, RFP expressing tachyzoites were cultured in presence of KU-55933 or DMSO, showing a significant reduction of tachyzoite growth in a dose-dependent manner and with IC_50_ = 2.49 μM (Figure 3C).

The presence of KU-55933 at these concentrations did not induce alterations in uninfected HFF monolayer morphology and MTT assay did not evidence impact of KU-55933 on HFF metabolism (Figure S2). In addition, KU-55933 did not disturb neither shape nor “rosette” organization of tachyzoites within PV (Figure 3D).

These results indicate that KU-55933 has a detrimental effect on intracellular tachyzoite replication. However, the indirect effect of PI3K inhibitors on tachyzoite replication due to HFF alterations, specifically at high doses, cannot be ruled out. To investigate if KU-55933 can have an effect directly on *Toxoplasma*, extracellular tachyzoites were incubated 4 h in presence of different doses of KU-55933 at room temperature. After that HFF monolayers were infected and incubated in absence of the drug for 12 h. Figure 3E shows a significant reduction in tachyzoite replication from 2.5 μM, suggesting that KU-55933 has a direct impact on *Toxoplasma*.

### KU-55933 Inhibits *Toxoplasma* H2A.X Phosphorylation at Serine 132 Under Fork Collapse

**Figure 4:**
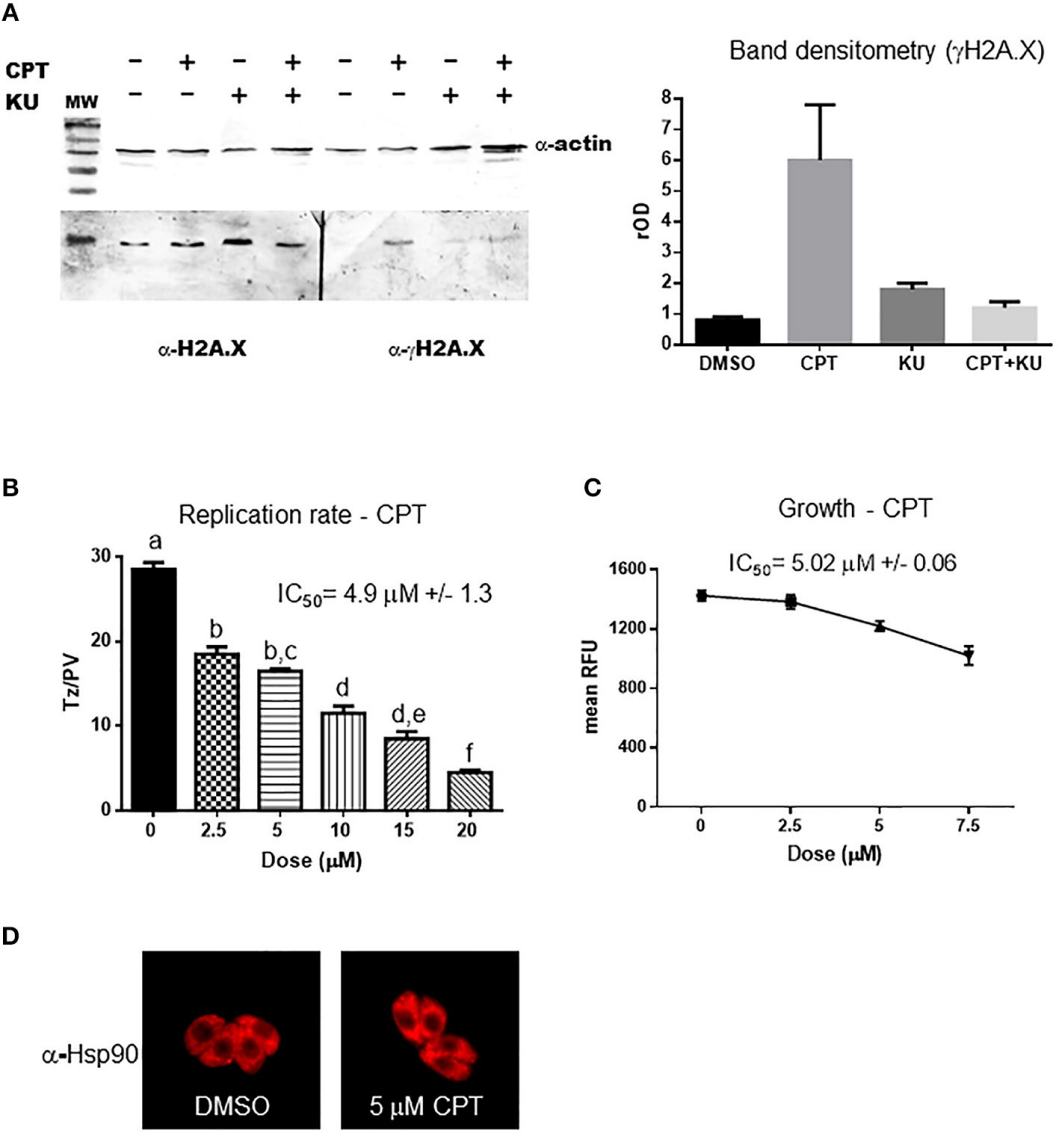
Camptothecin effect on H2A.X phosphorylation and tachyzoite replication. **(A)** Extracts of intracellular tachyzoite treated with 5 μM CPT, 5 μM KU-55933, both or DMSO for 24 h were analyzed by Western blot with anti-γH2AX antibody (α-γH2AX) and anti-*Toxoplasma* H2A.X. Anti-actin (α-actin) antibody was used as control of loaded protein. Band density was measured and relativized to DMSO (rOD). The graph is representative of three independent experiments with similar results. **(B)** Tachyzoites per PV (Tz/PV) were counted in 100 PV. Statistical analysis was performed by one-way ANOVA and Tukey's Multiple Comparison Test. Results are mean of three replicates plus SD. Same letters above the column indicate no significant differences; different letters indicate statistically significant differences between columns (*p* ≤ 0.05), according to one-way ANOVA and Tukey's multiple comparison test. Details: *p* ≤ 0.0001: a vs. b, c, d, e, and f; *p* ≤ 0.001: b vs. d; *p* ≤ 0.0001: b vs. e and f; *p* ≤ 0.01: c vs. d; *p* ≤ 0.0001: c vs. e and f; *p* ≤ 0.001: d vs. f; *p* ≤ 0.05: e vs. f. The graph is representative of three independent experiments with similar results. **(C)** Intracellular tachyzoites (RH RFP strain) were cultured in presence of DMSO or different doses of CPT in 96-well plates for 4 days and then read at 544 nm (bottom of the plate) in a microplate reader. Results are means of six replicates plus SD. **(D)** Arrangement of tachyzoites inside PV is visualized at 5 μM CPT. In presence of DMSO or the drug the typical rosette organization could be observed. PV with similar number of tachyzoites were selected to compare.

During cell replication DNA is duplicated in the S-phase, and replication forks remain stable until completion of DNA duplication. However, replication forks are subject to a variety of insults (dNTP depletion, DNA damage, DNA secondary structures, among others) that lead to fork stalling. The presence of several ssDNA and/or regressed forks (a structure also named “chickenfoot” in which complementary daughter ssDNAs regress and pair between them) promotes the collapse of forks and DSB (Postow et al., 2001; Alexander and Orr-Weaver, 2016). Camptothecin (CPT) is a topoisomerase I inhibitor that generates fork collapse, producing DSB and therefore γH2A.X, and induction of the HRR pathway (Chanoux et al., 2009; Xu et al., 2015; Rybak et al., 2016). We used CPT to further analyze KU-55933 activity, using the generation of γH2A.X in *Toxoplasma* as a marker of DSB in genomic DNA of tachyzoites. As it is known, ATM is able to phosphorylate SQ/TQ motif (Weber and Ryan, 2015) which is present in *T. gondii* H2A.X (SQEF) and detected by commercial γH2A.X (Dalmasso et al., 2009; Vonlaufen et al., 2010). Despite we purified *T. gondii* tachyzoites through 3 μm nitrocellulose filters before Western blot analysis, we tested the possibility to detect any contamination of HFF host cell. As observed in Figure S3, in our conditions anti-γH2A.X only detected a band in *T. gondii* lysate but not in HFF (Figure S3), suggesting that the experiment avoids putative false results due to the presence of HFF γH2A.X. The treatment with CPT increases the presence of γH2A.X in *Toxoplasma* as analyzed by Western blot (Figure 4A). The treatment of intracellular tachyzoites with KU-55933 did not block basal levels of γH2A.X, but the presence of KU-55933 in combination with CPT reduced γH2A.X levels compared parasites treated with CPT (Figure 4A).

CPT treatment of infected HFF showed an inhibition of parasite replication rate and growth in a dose-dependent manner with IC_50_ = 4.9 and 5.02 μM, respectively, (Figures 4B,C). CPT at these concentrations did not induce morphological alterations in uninfected HFF monolayers but a strong reduction of HFF metabolism was observed by MTT assay from 2.5 μM (Figure S2). However, the addition of 5 μM CPT did not disturb neither shape nor “rosette” organization of tachyzoites within PV (Figure 4D).

Our findings show that CPT generates DSB in the *Toxoplasma* genome, as evidenced by the increase in γH2A.X. The fact that this phosphorylation event could be abolished by the inhibitor KU-55933 during DNA damage suggests that it is mediated by TgATM kinase.

### Effect of CPT, Methyl Methane Sulfonate (MMS) and Hydroxyurea (HU) in Combination With KU-55933 on Tachyzoites

**Figure 5:**
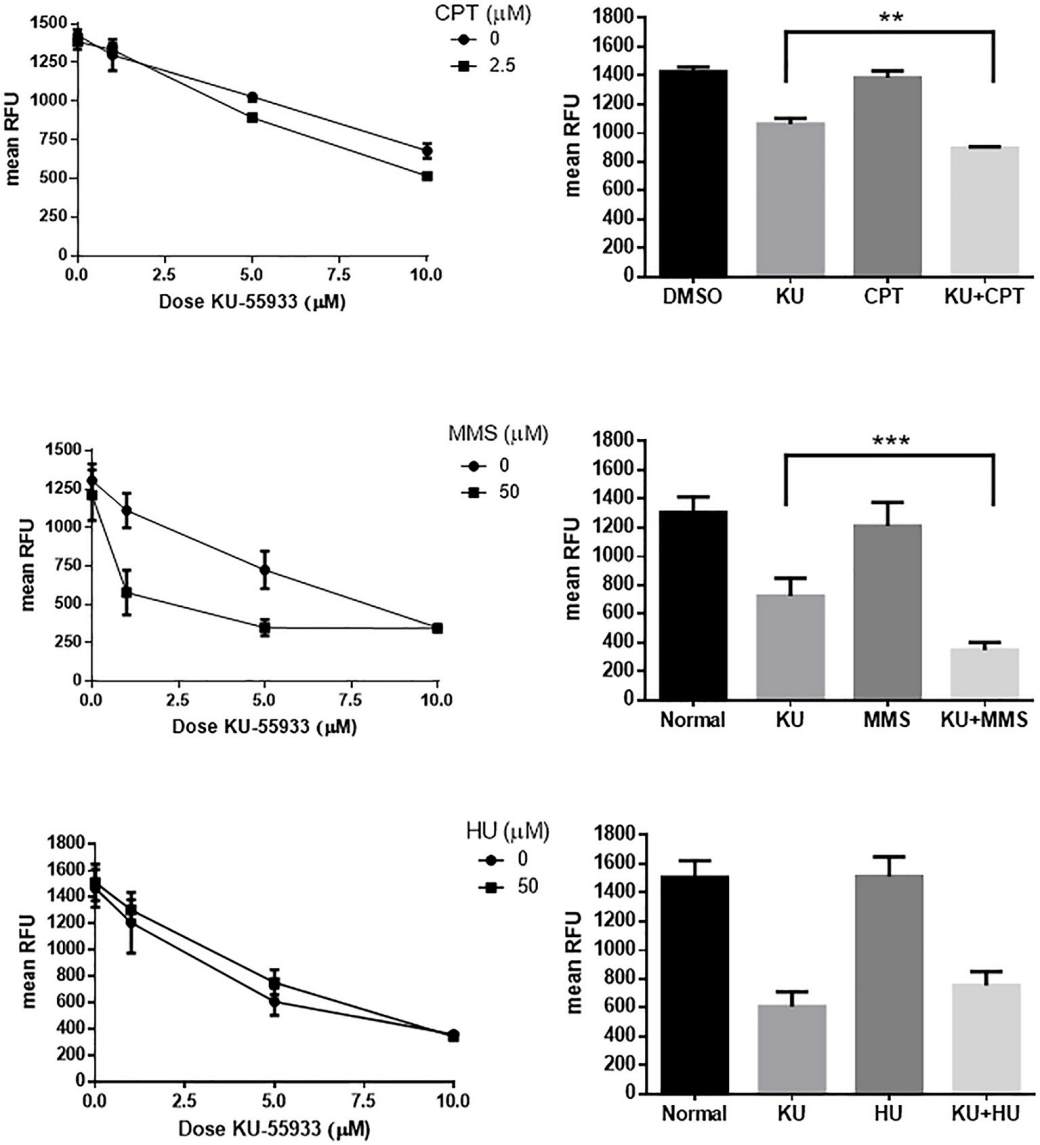
Effect on *Toxoplasma* ATM kinase with different DNA damaging agents. Intracellular tachyzoites (RH RFP strain) were treated with 2.5 μM CPT, 50 μM MMS, or 50 μM HU alone or in combination with KU-55933 at different doses during 96 h and their growth analyzed at 544 nM. As control DMSO (CPT) or PBS (MMS and HU) were used. Results were plotted by GraphPad Prism 6 (left panels). Combination of 5 μM KU-55933 and 2.5 μM CPT, 50 μM MMS, and 50 μM HU, and controls, were plotted as bar graphs (right panels). Statistical analysis was performed by one-way ANOVA and Tukey's Multiple Comparison Test. Results are mean of three replicates plus SD. There were not significant differences between DMSO and CPT, normal and MMS or normal and HU. Only differences between KU-55933 and combination is shown. **p ≤ 0.01; ***p ≤ 0.001, according to one-way ANOVA and Tukey's multiple comparison test. The graph is representative of three independent experiments with similar results.

In order to test the effect of other DNA damaging agents on *Toxoplasma* replication, we analyzed methyl methane sulfonate (MMS) and hydroxyurea (HU) (de Melo et al., 2000; Vonlaufen et al., 2010) using *Toxoplasma* RFP parasites, treated alone or in combination with KU-55933. A dose of 2.5 μM CPT in combination with KU-55933 was also analyzed. HU and MMS both block tachyzoite replication at concentrations higher than 50 μM (Figure S4). KU-55933 treatment administered with CPT or MMS increased the inhibitory effect of KU-55933 whereas HU in combination with KU-55933 presented no synergistic effect (Figure 5).

## Discussion

In this study, we demonstrated that PI3K inhibitors such as caffeine and KU-55933 are able to block *Toxoplasma* tachyzoite replication. A previous study has shown that caffeine, as an agonist of ryanodine-responsive calcium-release channels, increased the level of intracellular Ca^2+^ in *Toxoplasma* (Chini et al., 2005). In our study, we found that caffeine also produces a strong effect on intracellular tachyzoite replication. CGK 733, an ATM/ATR kinases inhibitor, has been shown to block *Toxoplasma* tachyzoite growth in a recent small molecule screen (Dittmar et al., 2016). These collective studies suggest that PI3 kinases, including ATM/ATR kinases, are important modulators for parasite growth and replication, and thus serve as attractive drug targets.

Whereas, caffeine targets a broad range of kinases and phosphatases (Velic et al., 2015), KU-55933 is specific for human ATM kinase (IC_50_ = 12.9 nM) being able to inhibit DNA-PK and ATR at IC_50_ = 2.5 and 16.6 μM, respectively (Hickson et al., 2004). KU-55933 blocks tachyzoite replication and generates G1-phase arrest, suggesting that TgATM kinase may have a role along *Toxoplasma* cell cycle. ATM kinase has a large number of substrates associated with the DDR, especially those involved in DSB repair (Matsuoka et al., 2007). Interestingly, the effect of KU-55933 on tachyzoite replication and growth was observed without any exogenous DNA damage treatment, suggesting that ATM kinase is required during tachyzoite cell cycle. Since ATM kinase is a key kinase that triggers the DDR during checkpoints when DSBs are present in DNA, it is possible that the demands of rapid tachyzoite replication create DNA replication stress and fork collapse generating one-ended DSB, similar to what is observed in cancer cells (Hickson et al., 2004; Alexander and Orr-Weaver, 2016; Zhang et al., 2016). Recently, it was observed that DDR associated with ATM kinase and histone ubiquitination is required for proper DNA replication in cells without S-phase perturbation (Schmid et al., 2018). The presence of basal γH2A.X is consistent with this conclusion.

When studying the effects of drugs on intracellular parasites, it is hard to rule out their potential effect on the host cells. One way to address this issue is to treat extracellular parasites with the drugs prior to infecting host cells. For example, the treatment of human retinal pigment epithelial cells, ARPE-19 with different PI3K inhibitors such as LY294002, wortmannin, GDC-0941, and ZSTK474, during 1 h prior to *T. gondii* infection blocked tachyzoite replication by reducing activation of host AKT (Zhou et al., 2013). We found that pre-incubation of extracellular tachyzoite with KU-55933 led to a significant reduction of tachyzoite replication following infection of HFFs. This result suggests KU-55933 can act directly on TgATM kinase and impede its ability to function during infection. Since extracellular tachyzoite is not a replicative stage, the effect of KU-55933 at this stage is intriguing. One explanation could be that KU-55933 is affecting the fitness of extracellular tachyzoites that need to recover after invasion. In this sense, ATM kinase has also been described to have a role in peroxisomes activating some proteins in response to reactive oxygen species (ROS), among them TORC1 (Alexander et al., 2010; Ditch and Paull, 2012; Zhang et al., 2015). Another explanation may be that treated tachyzoites contains residual traces of KU-55933 after host cell entry, requiring a time for ATM kinase recovery, and its participation in DNA replication process. Further analysis should be done to shed light on this question.

Recently, Dittmar et al. (2016) screened 1,120 compounds for an effect against *Toxoplasma* growth; in their study, KU-55933 at 5 μM showed no inhibitory effect, contrasting with our results. We found that the IC50 for KU-55933 against *Toxoplasma* was 2.15 μM, a concentration below the usual dose (10 μM) that produces an effect on mammalian cells (Teng et al., 2015; Tian et al., 2015). As ATM kinase is a known target of KU-55933, our results are in agreement with a genome-wide CRISPR screen suggesting that TgATM kinase is essential for tachyzoite viability (Sidik et al., 2016). Importantly, treatment of tachyzoites with KU-55933 impairs H2A.X phosphorylation, indicating that TgATM kinase is sensitive to KU-55933 during DDR.

Our observations indicate that CPT is able to generate DSB damage on parasite DNA, probably during tachyzoite replication, since it induces an increase of γH2A.X. Our findings lend support to the idea that DNA topoisomerases may also be promising drug targets in Apicomplexan and trypanosomatid parasites (Garcia-Estrada et al., 2010; D'Annessa et al., 2015). However, in our conditions CPT induced a decay in HFF metabolism as measured by MTT assay, suggesting certain toxic effect on host cell. Interestingly, this toxicity did not impair tachyzoite replication inside the host cell, but could affect our interpretation of data relative to blocking *T. gondii* replication. Recently, a novel plasmodial topoisomerase I venom was designed on CPT derivative topotecan structure (Cortopassi et al., 2014). They demonstrated that a compound named LQB223 has a high selectivity for *P. falciparum* topoisomerase I in comparison with human counterpart and reduced *Plasmodium berghei* parasitemia in mice. In the future, a selective *T. gondii* topoisomerase I venom should be analyzed to confirm the value of this therapeutic strategy.

Interestingly, here we demonstrate in a first approach that KU-55933 could have a synergic effect when used in combination with DNA damaging agents such as MMS and CPT, even at low doses. This could be due to the effect of these compounds generating DSB combined with the inhibition of DSB repair by KU-55933. This strategy, opens the possibility to investigate the value of druggable HRR and DNA replication factors. As mentioned above, in the future, a similar strategy could be used but using a *Toxoplasma* specific topoisomerase I venom, possibly LQB223, which could be used in combination with KU-55933, analogs or HRR inhibitors (e.g., Mre11 targets) that are being tested in human.

HU is known to generate fork stalling and activate DDR via ATR kinase rather than ATM kinase (Abraham, 2001), which may explain lack of synergy when combining it with KU-55933. In fact, it was observed that at low doses (50 μM), HU cannot present synergic effect with KU-55933 as observed at high doses (e.g., 1 mM) in mammal cells, in which a ATM-associated G1/S-phase arrest is occurring (Snyder et al., 2009).

In summary, we identified drugs effective in producing DSB in the parasite, and others that affect the mechanisms of DDR. Our findings imply that the mechanisms of DSB repair, for example the HRR pathway that repairs DSBs during DNA replication, could be replete with novel therapeutic targets to combat toxoplasmosis.

## Author Contributions

JM and AG accomplished most of the assays and the analysis, equally. SB contributed with the standarization of the caffeine, CPT, and KU-55933 experiments. DM performed experiments about Toxoplasma cell cycle analysis. DR contributed with the citotoxicity analysis. WS, LV, and SA contributed with the direction, analysis of the data, and writing the manuscript.

### Conflict of Interest Statement

The authors declare that the research was conducted in the absence of any commercial or financial relationships that could be construed as a potential conflict of interest.
