# Supplementary material for: Evaluation of ATM Kinase Inhibitor KU-55933 as Potential Anti-Toxoplasma gondii Agent
Source: Front Cell Infect Microbiol. 2019 Feb 13;9:26. doi: 10.3389/fcimb.2019.00026 (PMC6381018; doi:10.3389/fcimb.2019.00026)
Supplement: Supplementary file 1 [file Image_1.pdf]

A

```
                       10        20        30        40        50        60        70
              ....|....|....|....|....|....|....|....|....|....|....|....|....|....|
TgATM         ----------------------------------------------------------------------
NCLIV_064490  ----------------------------------------------------------------------
HuATM         MSLVLNDLLICCRQLEHDRATERKKEVEKFKRLIRDPETIKHLDRHSDSKQGKYLNWD----AVFRFLQK
ScTel1        --MEDHGIVETLNFLSSTKIKERNNALDELTTILKEDPERIPTKALSTTAEALVELLASEHTKYCDLLRN

                       80        90       100       110       120       130       140
              ....|....|....|....|....|....|....|....|....|....|....|....|....|....|
TgATM         ----------------------------------------------------------------------
NCLIV_064490  ----------------------------------------------------------------------
HuATM         YIQKETECLRIAKPNVSASTQASRQKKMQEISSLVKYFIKCANRRAPRLKCQELLNYIMDTVKDSSNGAI
ScTel1        LTVSTTNKLSLSENRLSTISYVLRLFVEKSCERFKVKTLKLLLAVVPELMVKDGSKSLLDAVSVHLSFAL

                      150       160       170       180       190       200       210
              ....|....|....|....|....|....|....|....|....|....|....|....|....|....|
TgATM         ----------------------------------------------------------------------
NCLIV_064490  ----------------------------------------------------------------------
HuATM         YGADCSN------ILLKDILSVRKYWCEISQQQWLELFSVYFRLYLKPSQDVHRVLVARIIHAVTKGCCS
ScTel1        DALIKSDPFKLKFMIHQWISLVDKICEYFQSQMKLSMVDKTLTNFISILLNLLALDTVGIFQVTRTITWT

                      220       230       240       250       260       270       280
              ....|....|....|....|....|....|....|....|....|....|....|....|....|....|
TgATM         ----------------------------------------------------------------------
NCLIV_064490  ----------------------------------------------------------------------
HuATM         QTDGLNSKFLDFFSKAIQCARQEKSSSGLNHILAALTIFLKTLAVNFRIRVCELGDEILPTLLYIWTQHR
ScTel1        VIDFLRLSKKENGNTRLIMSLINQLILKCHCFSVIDTLMLIKEAWSYNLTIGCTSNELVQDQLSLFDVMS

                      290       300       310       320       330       340       350
              ....|....|....|....|....|....|....|....|....|....|....|....|....|....|
TgATM         ----------------------------------------------------------------------
NCLIV_064490  ----------------------------------------------------------------------
HuATM         LNDSLKEVIIELFQLQIYIHHPKGAKTQEKGAYESTKWRSILYNLYDLLVNEISHIGSRGKYSSGFRNIA
ScTel1        S-----ELMNHKLPYMIGQENYVEELRSESLVSLYREYILLRLSNYKPQLFTVNHVEFSYIRGSRDKNSW

                      360       370       380       390       400       410       420
              ....|....|....|....|....|....|....|....|....|....|....|....|....|....|
TgATM         ----------------------------------------------------------------------
NCLIV_064490  ----------------------------------------------------------------------
HuATM         VKENLIELMADICHQVFNEDTRSLEISQSYTTTQRESSDYSVPCKRKKIELGWEVIKDHLQKSQNDFDLV
ScTel1        FALPDFRLRDRGGRSVWLKILGITKSLLTYFALNRKNENYSLLFKRRKCDS---DIPSILRISDDMDTFL

                      430       440       450       460       470       480       490
              ....|....|....|....|....|....|....|....|....|....|....|....|....|....|
TgATM         --------------------MIFGSSSFVSPASRSLSHADHLAAAGMCMKALQFVRVYVSQVAGGAAPGI
NCLIV_064490  ----------------------------------------------------------------------
HuATM         PWLQIATQLISKYPASLPNCELSPLLMILSQLLPQQRHGERTPYVLRCLTEVALCQDKRSNLESSQKSDL
ScTel1        IHLLEENSSHEFEVLGLQLCSFYGTLQDFTKSFAEQLKELLFSKFEKIQCFNWVCFSFIPLLSQKECELS

                      500       510       520       530       540       550       560
              ....|....|....|....|....|....|....|....|....|....|....|....|....|....|
TgATM         LRSRPKKEAKGPKARKKRETEDAKLADTKANVQCG--AGDTD------ACVKGDKTDADEACGEEAKNER
NCLIV_064490  ----------------------------------------------------------------------
HuATM         LKLWNKIWCITFRGISSEQIQAENFGLLGAIIQGS--LVEVDREFWKLFTGSACRPSCPAVCCLTLALTT
ScTel1        NGDMARLFKVCLPLVKSNESCQLSCLLLANSIKFSKQLLSDEKTINQIYDLYELSDILGPILVTNESFML
```

```
                     570       580       590       600       610       620       630
             ....|....|....|....|....|....|....|....|....|....|....|....|....|....|
TgATM        VEGRQTHSTEISVEAYATSRGEAVMPAKERFACLERRRSSGGKGRACGRSEMETKAGAPTTSTALGVKRE
NCLIV_064490 ----------------------------------------------------------------------
HuATM        SIVPGTVKMGIEQNMCEVNRSFSLKESIMKWLLFYQLEGDLENSTEVPPILHSNFPHLVLEKILVSLTMK
ScTel1       WGYLQYVGKDFQSMNGISSADRIFEWLKSKWNQLRGTDAKQDQFCNFISWLGNKYDPENPFNDKKGEGAN

                     640       650       660       670       680       690       700
             ....|....|....|....|....|....|....|....|....|....|....|....|....|
TgATM        GPADPQKPRQTHPFFQRRGQCKAPVTS--------EASVGRIPNHEDCSDISADFCIPQKRPNEVAGIRE
NCLIV_064490 ----------------------------------------------------------------------
HuATM        NCKAAMNFFQSVPECEHHQKDKEELSFSEVEELFLQTTFDKMDFLTIVRECGIEKHQSSIGFSVHQNLKE
ScTel1       PVSLCWDESHKIWQHFQEQREFLLGVKPEEKSECFNTPFFNLPKVSLDLTRYNEILYRLLENIESDAFSS

                     710       720       730       740       750       760       770
             ....|....|....|....|....|....|....|....|....|....|....|....|....|
TgATM        PSRSCSRSFPPTDGASSAWTADVSFSPLASLGLLRQGLGGDMLDNFLHFWSALDLQAIAFVALQLGCPVD
NCLIV_064490 ----------------------------------------------------------------------
HuATM        SLDRCLLGLSEQLLNNYSSEITNSETLVRCSRLLVGVLGCYCYMGVIAEEEAYKSELFQKAKSLMQCAGE
ScTel1       PLQKFTWVAKLIQIVDNLCGDSTFSEFIAAYKRTTLITIPQLSFDSQNSYQSFFEEVLSIRTINVDHLVL

                     780       790       800       810       820       830       840
             ....|....|....|....|....|....|....|....|....|....|....|....|....|
TgATM        ALIFAEQELERRCPDSGLEEGLAVVSQQWQRAQWDRMPQIRS---VQVLRQMLLEAQRGQGRGPDEVPRP
NCLIV_064490 ----------------------------------------------------------------------
HuATM        SITLFKNKTNEEFRIGSLRNMMQLCTRCLSNCTKKSPNKIASGFFLRLLTSKLMNDIADICKSLASFIKK
ScTel1       DKINMKEIVNDFIRMQKNKSQTGTSAINYFEASSEDTTQNNSPYTIGGRFQKPLHSTIDKAVRAYLWSSR

                     850       860       870       880       890       900       910
             ....|....|....|....|....|....|....|....|....|....|....|....|....|
TgATM        PRTVANAAPDQEEASGGKGGAAEGGTDEQGTWEKMEGVAEGAGRALD-----------------------
NCLIV_064490 ----------------------------------------------------------------------
HuATM        PFDRGEVESMEDDTNGNLMEVEDQSSMNLFNDYPDSSVSDANEPGESQSTIGAINPLAEEYLSKQDLLFL
ScTel1       NKSISERLVAILEFSDCVSTDVFISYLGTVCQWLKQAIGEKSSYNKILEEFTEVLG--------------

                     920       930       940       950       960       970       980
             ....|....|....|....|....|....|....|....|....|....|....|....|....|
TgATM        --------CGHVVPGDKGKGEEGTVGSDGNRPSKEQRMSPESMRHFQLSLQRLREMA-----IRPANASA
NCLIV_064490 -----------------------------------------------MALQRLREVS-----VRPANASA
HuATM        DMLKFLCLCVTTAQTNTVSFRAADIRRKLLMLIDSSTLEPTKSLHLHMYLMLLKELPGEEYPLPMEDVLE
ScTel1       -EKLLCNHYSSSNQAMLLLTSYIEAIRPQWLSYPEQPLNSDCNDILDWIISRFEDNSFTGVAPTVNLSML

                     990      1000      1010      1020      1030      1040      1050
             ....|....|....|....|....|....|....|....|....|....|....|....|....|
TgATM        LLTPVG---------------------------------------PAPLFLLVSQALINANEE-------
NCLIV_064490 LLTPVG---------------------------------------PAPLFLLVSQALINANEE-------
HuATM        LLKPLSNVCSLYRRDQDVCKTILNHVLHVVKNLGQSNMDSENTRDAQGQFLTVIGAFWHLTKERKYIFSV
ScTel1       LLSLLQN---------------------------HDLSHGSIRGGKQRVFATFIKCLQKLDSS-------

                    1060      1070      1080      1090      1100      1110      1120
             ....|....|....|....|....|....|....|....|....|....|....|....|....|
TgATM        --------DLSAVTDCLSPWHTSNFLIQRMHAENDWLATLTLQQEQLETLEAKRLQVLAQLRQLHST---
NCLIV_064490 --------DLSAVTDCLSAWHTSNFLIQRMHSDNDWLATLTLQQEQLRNLESERAQLGAQLRQSRTS---
HuATM        RMALVNCLKTLLEADPYSKWAILNVMGKDFPVNEVFTQFLADNHHQVRMLAAESINRLFQDTKGDSSRLL
ScTel1       --------NIINIMNSISSYMAQVSYKNQSIIFYEIKSLFGPPQQSIEKSAFYSLAMSMLSLVSYPS-LV

                    1130      1140      1150      1160      1170      1180      1190
             ....|....|....|....|....|....|....|....|....|....|....|....|....|
TgATM        -GLHGHPDQAAWSASSIGLEARS-VFENEAVGRSRITQTGDR------------------------DLHT
NCLIV_064490 -DLDFPAVQADWSVASLTVEANG-NLDAADAGRSPVARQRRR------------------------LLRT
HuATM        KALPLKLQQTAFENAYLKAQEGMREMSHSAENPETLDEIYNRKSVLLTLIAVVLSCSPICEKQALFALCK
ScTel1       FSLEDMMTYSGFNHTRAFIQQALNKITVAFRYQNLTELFEYCK-----------------------FDLI
```

```
                 1200      1210      1220      1230      1240      1250      1260
             ....|....|....|....|....|....|....|....|....|....|....|....|....|....|
TgATM        RLREVEEELYATKLQMADALGAIGLHSLRESLLVSLSLSSSVPSQLSHSLSYPS----------------
NCLIV_064490 RLREVEEQLYATKIQMADALGIIGLHSLRESLLASLRFSSSA-SPLPYTPAYPS----------------
HuATM        SVKENGLEPHLVKKVLEKVSETFGYRRLEDFMASHLDYLVLEWLNLQDTEYNLSSFPFILLNYTNIEDFY
ScTel1       MYWFNRTKVPTSKLEKEWDISLFGFADIHEFLGRYFVEISAIYFSQGFNQKWILDMLHAITGNGDAYLVD

                 1270      1280      1290      1300      1310      1320      1330
             ....|....|....|....|....|....|....|....|....|....|....|....|....|....|
TgATM        ---LACTLSSQRSPMDFGGSWSGP-----------GGGALG-GLGTRPFSASIGVGFPPLLED-EGKETD
NCLIV_064490 ---WSSTLSFHCSPPRFQGIWCRA-----------GGADPSTAAGTRPLWASIRETCTRFGEDREDAGAD
HuATM        RSCYKVLIPHLVIRSHFDEVKSIANQIQEDWKSLLTDCFPKILVNILPYFAYEGTRDSGMAQQRETATKV
ScTel1       NSYYLCIPLAFISGGVNELIFDILPQIS-------GKTTVKYHKKYRLLMLKWIIRFTDLGSLTELRSTV

                 1340      1350      1360      1370      1380      1390      1400
             ....|....|....|....|....|....|....|....|....|....|....|....|....|....|
TgATM        CG---ATAWLEKKFECLWR-------------------------------------------LHRWDC
NCLIV_064490 CGES-SRAWLEKKFECLWR-------------------------------------------LHKWDC
HuATM        YDMLKSENLLGKQIDHLFISNLPEIVVELLMTLHEPANSSASQSTDLCDFSGDLDPAPNPPHFPSHVIKA
ScTel1       EKLFPTSYLSPYLFENSSVS---------------------------------------------MRYQY

                 1410      1420      1430      1440      1450      1460      1470
             ....|....|....|....|....|....|....|....|....|....|....|....|....|....|
TgATM        DET-PWGASQTEICPMFDSNADLTESFSPCPRRSRDQTQPSLSLFN------------------------
NCLIV_064490 GETTPLGASRTEPWSSLDSGGEQRDSFSPCPPDSCGETGQNLSLFN------------------------
HuATM        TFAYISNCHKTKLKSILEILSKSPDSYQKILLAICEQAAETNNVYKKHRILKIYHLFVSLLLKDIKSGLG
ScTel1       PLHIPLALGATLVQTQFAHEKNNTHEFKLLFLSVITDLEKTSTYIGKLRCAR------------------

                 1480      1490      1500      1510      1520      1530      1540
             ....|....|....|....|....|....|....|....|....|....|....|....|....|....|
TgATM        --------GCIYATLSLIHGAASCHPSSLVCPLQSSQACSKG-----------ARASHSATCH-------
NCLIV_064490 --------GCIYSTLSLIHGAASCRPSSSLPPRASSASQTSNSDVRALPSCLEGRAPHVAAFR-------
HuATM        GAWAFVLRDVIYTLIHYINQRPSCIMDVSLRSFSLCCDLLSQVCQTAVTYCKDALENHLHVIVGTLIPLV
ScTel1       ------ELKYLFVLYENVLVKSSTLNFIIIRLSKFLIDTQIHDEVITIFSSLLNLADKNTFEIEPSLPN-

                 1550      1560      1570      1580      1590      1600      1610
             ....|....|....|....|....|....|....|....|....|....|....|....|....|....|
TgATM        -----QQKSLFSTPEDVIVDAGADA-------------------------GNGDQVDG-----EAEEAL
NCLIV_064490 -----RHTRHCAVPTTGTLEGDDED-------------------------DEDEQGDTYDEAEELDHGA
HuATM        YEQVEVQKQVLDLLKYLVIDNKDNENLYITIKLLDPFPDHVVFKDLRITQQKIKYSRGPFSLLEEINHFL
ScTel1       ---LFCKIFIYLRENKQLSPSFQQAIKLLEHRDLIKIKTWKYCLDAIFGNIVQDDIYENTELLDASDCGV

                 1620      1630      1640      1650      1660      1670      1680
             ....|....|....|....|....|....|....|....|....|....|....|....|....|....|
TgATM        SFQRGLSSTMARLRAVQTSRHALALALERLARSPQLADS------------------------------SL
NCLIV_064490 TSQRVFNTILTRLRAVQTSRHALAIALERLASTAQSASS------------------------------AV
HuATM        SVSVYDALPLTRLEGLKDLRRQLELHKDQMVDIMRASQDNPQDGIMVKLVVNLLQLSKMAINHTGEKEVL
ScTel1       DDVVLVSLLFSYARRPVASKIGCSLSKAAAINILKHHVPKEYLSKN-------------------FKLWF

                 1690      1700      1710      1720      1730      1740      1750
             ....|....|....|....|....|....|....|....|....|....|....|....|....|....|
TgATM        PSIGHSAVDAQDQSLSRRLLGAYVELSMAHALQRATTLNLLLNQCEQDWNDDPCGAASFEHLQSSWANDL
NCLIV_064490 SSFGPSSANAPDPSLSRRLLSAYVELSMAHALQLATTLNLRLHQGEP-CQDAQC--DAFEHLQSSWANDL
HuATM        EAVGSCLGEVGPIDFSTIAIQHSKDASYTKALKLFEDKELQWTFIMLTYLNNTLVEDCVKVRSAAVTCLK
ScTel1       AALSRRILQQEVQRERSTNFNNEVHLKNFEMVFRHPEQPHMIYQRISTFNKEAELYDSTEVFFISECILT
```

```
                     1760      1770      1780      1790      1800      1810      1820
              ....|....|....|....|....|....|....|....|....|....|....|....|....|....|
TgATM         AVAAGAASACSSSASSSSLSSLPFFLEPLVSLETCILQAAIP----------------------------
NCLIV_064490  AVAAGPPAACSSAAASSPVSSLPFFLEPLVSLQTSILEAAIP----------------------------
HuATM         NILATKTGHSFWEIYKMTTDPMLAYLQPFRTSRKKFLEVPRFDKENPFEGLDDINLWIPLSENHDIWIKT
ScTel1        YLVGYSIGNSESEFCFR-DNIMNENKDKVAPLDKDVLNAIYPLANNFGMESFICDTYLSVNEPYNCWLSK

                     1830      1840      1850      1860      1870      1880      1890
              ....|....|....|....|....|....|....|....|....|....|....|....|....|....|
TgATM         -------------------------------SFHCIPSSAPFLPRSFETWTLREASGAAACMRERQEDTL
NCLIV_064490  -------------------------------SFHIYPR-APCSGEFCEHWAPRGTHAGTGSVHD-QQATR
HuATM         LTCAFLDSGGTKCEILQLLKPMCEVKTDFCQTVLPYLIHDILLQDTNESWRNLLSTHVQGFFTSCLRHFS
ScTel1        FAR---------------------------SLIHQISFNIPPIVCLYPLCKGSTAFCELVLTDLFFLSTT

                     1900      1910      1920      1930      1940      1950      1960
              ....|....|....|....|....|....|....|....|....|....|....|....|....|....|
TgATM         RFQRCCGDRGEALTDAHACGCHVRRPAARHLLLLSQMKRER-----------------------------
NCLIV_064490  GTERRRRDR-IELEGARACGCHVRRPAARHLLFLAEMKRER-----------------------------
HuATM         QTSRSTTPANLDSESEHFFRCCLDKKSQRTMLAVVDYMRRQKRPSSGTIFNDAFWLDLNYLEVAKVAQSC
ScTel1        YDPKSCLNWSNRIFTQIAMLLHVKDSEIKLKMLFNVIKMIRMG-------------------SRCKERNC

                     1970      1980      1990      2000      2010      2020      2030
              ....|....|....|....|....|....|....|....|....|....|....|....|....|....|
TgATM         -GDWRGSLENLLQAKEHLLQLQRDRERPFWEAVPAQLAVWRDAYPSGVSCHASDKDSLLLVSSLAAVPPV
NCLIV_064490  -GDWRGSLENLLQAKAHLLQLQRDRERPSWEAFP------------------------------------
HuATM         AAHFTALLYAEIYADKKSMDDQEKRSLAFEEGSQSTTISSLSEKSKEETGISLQDLLLEIYRSIGEPDSL
ScTel1        LRIYSSLDLQEICQISLKIKEFKFGYLLFEEMNMPNIREMNINTLQKIYECINDGDFLAGLPVPHSIEGV
                         FFFFFFFFFFFFFFFFFFFFFFFFFFFFFFFFFFFFFFFFFFFFFFFFFFFFFFFFFFF

                     2040      2050      2060      2070      2080      2090      2100
              ....|....|....|....|....|....|....|....|....|....|....|....|....|....|
TgATM         LSGLHGGRHLPPAFSPSFLCVDLF---LVQWQLAKTLYLAGDRSGSLNIAS--RLCQPKNWPVLPFQKAR
NCLIV_064490  ------------------------------WQLAKTLYLAGDRSGSLNIAS--RLCQPKNWPMLPFQNLN
HuATM         YGCGGGKMLQPITRLRTYEHEAMWGKALVTYDLETAIPSSTRQAGIIQALQNLGLCHILSVYLKGLDYEN
ScTel1        LNSINRIDSDTWKRFLFNNADFDANYTTSLEEEKESLIKATEDSGFYGLTSLLESRLSGSSDVYKWNLEL
              FFFFFFFFFFFFFFFFFFFFFFFFFFFFFFFFFFFFFFFFFFFFFFFFFFFFFFFFFFFFFFFFFFFFFF

                     2110      2120      2130      2140      2150      2160      2170
              ....|....|....|....|....|....|....|....|....|....|....|....|....|....|
TgATM         ------MSGRSDQSASSGIQGSAAVSGDAARSLGALKQPIQRLLPTGCDR-ENIAFETPKALLNGDMETL
NCLIV_064490  ------ATSAAGPSCATSCGGAGAPAAGDARPSHGCSPQASFKSHSFANR-TSSLWETKPVSRTEDLDGL
HuATM         KDWCPELEELHYQAAWRNMQWDHCTSVSKEVEGTSYHESLYNALQSLRDREFSTFYESLKYARVKEVEEM
ScTel1        GDWKLLTPKVVDSKAKGLYYAIKNLPQDVGFAEKSLEKSLLTIFDSRQHFISQTEWMDTLNAIIEFIKIA
              FFFFFFFFFFFFFFFFFFFFFFFFFFFFFFFFFFFFFFFFFFFFFFFFFFFFFFFFFFFFFFFFFFFFFF

                     2180      2190      2200      2210      2220      2230      2240
              ....|....|....|....|....|....|....|....|....|....|....|....|....|....|
TgATM         AVEKFR-----QAMIDALCSCGEWQFRGRWATEATVRRRYFSLALRLGSVSSSLEP--------------
NCLIV_064490  AVQKFR-----GAMIDALCSCGEWQFRGRWATEATVRRRYFSLALRLGPVSGSLEP--------------
HuATM         CKRSLESVYSLYPTLSRLQAIGELESIGELFSRSVTHRQLSEVYIKWQKHSQLLKDSDFSFQEPIMALRT
ScTel1        AIPQDVTSFP-QTLMSIMKADKERLNTIDFYDHKTTLKSRHTLMNVLSRNSLDENVKCS-----------
              FFFFFFFFFFFFFFFFFFFFFFFFFFFFFFFFFFFFFFFFFFFFFFFFFFFFFFFFFFFFFFFFFFFFFF

                     2250      2260      2270      2280      2290      2300      2310
              ....|....|....|....|....|....|....|....|....|....|....|....|....|....|
TgATM         ------------SQRLATLIDDQLQSYSDYKHSLPSLVMADLRRKEKAEIESLLQQLSAACEERGEE---
NCLIV_064490  ------------NQRLATLIDDQLQSYSDYKHSVPSLVMADLRRKEKAEIESLLQQLSSAFEERGEE---
HuATM         VILEILMEKEMDNSQRECIKDILTKHLVELSILARTFKNTQLPERAIFQIKQYNSVSCGVSEWQLEE---
ScTel1        ---------KYLRLGSIIQLANYVQLAIANGAPQDALRNATLMSKTVKNIAKLYDDPSVVSQIEKLASFT
              FFFFFFFFFFFFFFFFFFFFFFFFFFFFFFFFFFFFFFFFFFFFFFFFFFFFFFFFFFFFFFFFFFF
```

```
                       2320      2330      2340      2350      2360      2370      2380
             ....|....|....|....|....|....|....|....|....|....|....|....|....|....|
TgATM        -EQSRRAYAYKKFLQHHIDILMQK--QHGLEREREREYQERTQWALEAVT--LYATCLRDPFLPHATKTT
NCLIV_064490 -EQTRRAHAQQKFLQHHIDILLQK--QRVLEREREKEYQERTQWALEAVT--LYATCLKDPFLPHAAKTT
HuATM        -AQVFWAKKEQSLALSILKQMIKK--LDASCAANNPSLKLTYTECLRVCGNWLAETCLENPAVIMQTYLE
ScTel1       SANALWESREYKAPVMIMRDLLAQNEKNISESILYDDFKLLINVPMDQIKARLVKWSSESRLEPAAAIYE
              FFFFFFFFFFFFFFFFFFFFFFF  FFFFFFFFFFFFFFFFFFFFFFFFFFFFFFFFFFFFFFFFFFFF

                       2390      2400      2410      2420      2430      2440      2450
             ....|....|....|....|....|....|....|....|....|....|....|....|....|....|
TgATM        SRVLRLWF--EWGDSVPEMNAALHRLLIAPFASSQPQATQQVDSAVS-SKSSSAPTRKMHDDDGSTVEGP
NCLIV_064490 SRVLSLWF--EWGDDVPEMNAALHRLLIAPFTSAQPQHRQDAGHSSSPFDSSFSSAQRMNEEDDSRVGGP
HuATM        KAVEVAGN--YDGESSDELRNGKMKAFLSLARFSDTQYQRIENYMKSSEFENKQALLKRAKEEVGLLREH
ScTel1       KIIVNWDINVEDHESCSDVFYTLGSFLDEQAQKLRSNGEIEDREHRSYTGKSTLKALELIYKNTKLPENE
             FFFFFFFF  FFFFFFFFFFFFFFFFFFFFFFFFFFFFFFFFFFFFFFFFFFFFFFFFFFFFFFFFFFFF

                       2460      2470      2480      2490      2500      2510      2520
             ....|....|....|....|....|....|....|....|....|....|....|....|....|....|
TgATM        PDLRHLLPFVYQIASRLGSPAESPFQRTLRELLVTTARQYP-------------FQCLYQLVAIRNGRRI
NCLIV_064490 PDLRHLLPFVYQIASRLGSPAESPFQRTLRDLLVTTARQYP-------------FQCLYQLVAIRNGRRI
HuATM        KIQTNRYTVKVQRELELDELALRALKEDRKRFLCKAVENYINCLLSGEEHD-MWVFRLCSLWLENSGVSE
ScTel1       RKDAKRHYNRVLLQYNRDSEVLKALLLQKEKFLWHALHFYLNTLVFSNRYDNDIIDKFCGLWFENDDNSK
             FFFFFFFFFFFFFFFFFFFFFFFFFFFFFFFFFFFFFFFFFFFFFFFFFFF FFFFFFFFFFFFFFFFFF

                       2530      2540      2550      2560      2570      2580      2590
             ....|....|....|....|....|....|....|....|....|....|....|....|....|....|
TgATM        PPGHRGSDSFTVQQDKIDAAQEVLARIADSSPS--------LKFVVAAVESLVDFYNDLCLLDFDEEIR-
NCLIV_064490 PPGHRGADSFTVQQDKIDAAQDVLGRIADSSPA--------LKFVVAAVECLVDFYNDLCLLDFDDETR-
HuATM        VNGMMKRDGMKIPTYKFLPLMYQLAARMGTKMMGGLGFHEVLNNLISRISMDHPHHTLFIILALANANRD
ScTel1       INQLLYKEIGTIPSWKFLPWVNQIASKISMEEN---EFQKPLQLTMKRLLYKLPYDSLYSVMSILLYEK-
             FFFFFFFFFFFFFFFFFFFFFFFFFFFFFFFFFFFFFFFFFFFFFFFFFFFFFFFFFFFFFFFFFF

                       2600      2610      2620      2630      2640      2650      2660
             ....|....|....|....|....|....|....|....|....|....|....|....|....|....|
TgATM        ------DVQKRQRNLPLLQRGSSQSVRSILTPQEQMLRLPSFQKLEEYSSLLPVPTVELPRD--------
NCLIV_064490 ------TVQKKQRSLPLLQRGSSQSFPRRLTPQEQMIRLPSFQKLEEFSALLPIPTVALPRD--------
HuATM        EFLTKPEVARRSRITKNVPKQSSQLDEDRTEAANRIICTIRSRRPQMVRSVEALCDAYIILANLDATQWK
ScTel1       ------QSNKDTNISQKIQAVKKILLELQGYDRGAFAKKYLLPVQEFCEMSVELANLKFVQN-------T

                       2670      2680      2690      2700      2710      2720      2730
             ....|....|....|....|....|....|....|....|....|....|....|....|....|....|
TgATM        ---RLVPFQPPKTLSRIKNVPDFLLHLSSYIHSLHPCCLSGFP-GDRLRMRVTVIGSGITKPKLLCIWDR
NCLIV_064490 ---RLVPFQTPETLSRFKSAPDYLLHLSSYIHSLHPYCLSDFP-GDRLRMSVKVIGSGITKPKLLCIWDR
HuATM        TQRKGINIPADQPITKLKNLEDVVVPTMEIKVDHTGEYGNLVT-IQSFKAEFRLAG-GVNLPKIIDCVGS
ScTel1       KTLRLANLKIGQYWLKQLNMEKLPLPTSNFTVKSSADGRKARPYIVSVNETVGITTTGLSLPKIVTFNIS

                       2740      2750      2760      2770      2780      2790      2800
             ....|....|....|....|....|....|....|....|....|....|....|....|....|....|
TgATM        KGRKHKQCCKG-REDVRQDRVAQQLFSMLNQVFLEAPSTLEKNFYLRTYVVVPFSPAVGILEWVNDAVTL
NCLIV_064490 QGRKHKQCCKG-REDVRQDRVAQQLFSMLNQVFLEAPSTLEKNFYLRTYAVVPFSPAVGILEWVNDAVTL
HuATM        DGKERRQLVKG-RDDLRQDAVMQQVFQMCNTLLQRNTETRKRKLTICTYKVVPLSQRSGVLEWCTGTVPI
ScTel1       DGTTQKALMKGSNDDLRQDAIMEQVFQQVNKVLQNDKVLRNLDLGIRTYKVVPLGPKAGIIEFVANSTSL
                 KKKKKKK KKKKKKKKKKKKKKKKKKKKKKKKKKKKKKKKKKKKKKKKKKKKKKKKKKKKKKKKKK

                       2810      2820      2830      2840      2850      2860      2870
             ....|....|....|....|....|....|....|....|....|....|....|....|....|....|
TgATM        AQYLIGNPDNGYHGAHARYRPQDWTFTACRQKLRLAREKALAQRRQMLSLGTADDGGLQKGQQKKEASGA
NCLIV_064490 AQYLIGSPENGYHGAHARYRPQDWTFSACRHKLRLAREKALAQRRQLLSRGAGDDGAQQKGQQPTAVSRA
HuATM        GEFLVNNED----GAHKRYRPNDFSAFQCQKKMMEVQKKSFEEK--------------------------
ScTel1       HQILS-----------KLHTNDKITFDQARKGMKAVQTKSNEER--------------------------
             KKKKKKKKK    KKKKKKKKKKKKKKKKKKKKKKKKKKKKKKK
```

```
                       2880      2890      2900      2910      2920      2930      2940
              ....|....|....|....|....|....|....|....|....|....|....|....|....|....|
TgATM         DAGSHSLVESLVQTYDEICDHFKPVLHHFFLEHFPSADVWYEKRQRYRRTLAVSSMVSYIIGLGDRHTNN
NCLIV_064490  DMGPDPLVESLVQAYDEICGHFKPVLHYFFLEHFPSADAWYAQRQRYRRTLAVSSMVSYIIGLGDRHTNN
HuATM         -----------YEVFMDVCQNFQPVFRYFCMEKFLDPAIWFEKRLAYTRSVATSSIVGYILGLGDRHVQN
ScTel1        -----------LKAYLKITNEIKPQLRNFFFDSFPDPLDWFEAKKTYTKGVAASSIVGYILGLGDRHLNN
                         KKKKKKKKKKKKKKKKKKKKKKKKKKKKKKKKKKKKKKKKKKKKKKKKKKKKKKKKKKK

                       2950      2960      2970      2980      2990      3000      3010
              ....|....|....|....|....|....|....|....|....|....|....|....|....|....|
TgATM         ILLDVASGDLIHIDFGVVFEQGKLLAIPELVPFRLTRDLVDGLGCLGVAGCFKRDCETTMEVLRRVSPLV
NCLIV_064490  ILLDIASGDLIHIDFGVVFEQGKLLAVPELVPFRLTRDLVDGLGCLGVAGCFKRDCETTMEVLRRVSPLV
HuATM         ILINEQSAELVHIDLGVAFEQGKILPTPETVPFRLTRDIVDGMGITGVEGVFRRCCEKTMEVMRNSQETL
ScTel1        ILLDCSTGEPIHIDLGIAFDQGKLLPIPELVPFRLTRDIVDGFGVTGVDGLFRRSCERVYAVLRKDYVKV
              KKKKKKKKKKKKKKKKKKKKKKKKKKKKKKKKKKKKKKKKKKKKKKKKKKKKKKKKKKKKKKKKKKKKKK

                       3020      3030      3040      3050      3060      3070      3080
              ....|....|....|....|....|....|....|....|....|....|....|....|....|....|
TgATM         VSIVEVLLFDPLYRWCLDPRRILQDSDKSLALSPSSRPGSGPKKGEQKRGRDGCGRGRGKRTSVSTLLFA
NCLIV_064490  VSIVEVLLFDPLYRWCLDPRRILQESDKSLALAPS-RPGSDAKTGGPKRGRDATSRGRGPTSTTSTLPVA
HuATM         LTIVEVLLYDPLFDWTMNPLKALYLQQ----------------------------RPEDETELHPTLNAD
ScTel1        MCVLNILKWDPLYSWVMSPVKKY---------------------------------EHLFEEEHEITNFD
              KKKKKKKKKKKKKKKKKKCCCCCCCCC                             CCCCCCCCCCCCCCCC

                       3090      3100      3110      3120      3130      3140      3150
              ....|....|....|....|....|....|....|....|....|....|....|....|....|....|
TgATM         EQKGRKRGRHEAIPEAIPSSSAHHENGAAGAAEETRDHENSVDDVKRKHVDSEMPIVEDILTKAEGNMNA
NCLIV_064490  ERSRRKRGRLEAVPEQIPSGSALLDGGTKG--EAKRDPEEAVDEARGKQSAKDIPIVEDILTKAEGNMNA
HuATM         DQECKRN---------LSDIDQSFNKVAER----------------------------------------
ScTel1        NVS-----------KFISNNDRNENQESYR----------------------------------------
              CCCCCCC         CCCCCCCCCCCCCC

                       3160      3170      3180      3190      3200
              ....|....|....|....|....|....|....|....|....|....|..
TgATM         KLAVLTVRRKLEGYEEGEIAQLSVNAHVARLVNAAQDRTALAQMFVGWAPWV
NCLIV_064490  KRAVLTVRRKLEGYEEGEIAQLRVNAHVARLVNAAQDRTALAQMFVGWAPWV
HuATM         --VLMRLQEKLKGVEEG--TVLSVGGQVNLLIQQAIDPKNLSRLFPGWKAWV
ScTel1        --ALKGVEEKLMG------NGLSVESSVQDLIQQATDPSNLSVIYMGWSPFY
                CCCCCCCCCCCCCCC  CCCCCCCCCCCCCCCCCCCCCCCCCCCCCCCCC
```

B

Blastp analysis= Expect: 4e-72, Identities: 170/552(31%), Positives: 265/552(48%)

```
TgATM      LLPFVYQIASRLGSPAESP--FQRTLRELLVTTARQYPFQCLYQLVAIRNGRR-----IP  1591
            LP +YQ+A+R+G+         F   L  L+    +P    L+ ++A+ N   R       P
HuATM      FLPLMYQLAARMGTKMMGGLGFHEVLNNLISRISMDHPHHTLFIILALANANRDEFLTKP  1227
           FFFFFFFFFFFFFFFFFFFFFFFFFFFFFFFFFFFFFFFFFFFFFFFFFFFFF

TgATM      PGHRGS--------DSFTVQQDKIDAAQEVLARIADSSPSLKFVVAAVESLVDFYNDLCL  1643
              R S         S  + +D+ +AA  ++  I    P +    V +VE+L D Y  + L
HuATM      EVARRSRITKNVPKQSSQLDEDRTEAANRIICTIRSRRPQM---VRSVEALCDAY--IIL  1282

TgATM      LDFDEEIRDVQKRQRNLPLLQRGSSQSVRSILTPQEQMLRLPSFQKLEEYSSLLPVPTVE  1703
            + D       Q++  N+P  Q                    +   + LE+    + VPT+E
HuATM      ANLDATQWKTQRKGINIPADQP------------------ITKLKNLED----VVVPTME  1320
```

```
TgATM        LPRDRLVPFQPPKTLSRIKNVPDFLLHLSSYIHSLHPCCLSGFPGDRLRMRVTVIGSGIT  1763
             +  D    +    T+   K   +F L                               G+
HuATM        IKVDHTGEYGNLVTIQSFK--AEFRL-------------------------------AGGVN  1349

TgATM        KPKLLCIWDRKGRKHKQCCKGREDVRQDRVAQQLFSMLNQVFLEAPSTLEKNFYLRTYVV  1823
              PK++      G++ +Q  KGR+D+RQD V QQ+F M N +      T ++   + TY V
HuATM        LPKIIDCVGSDGKERRQLVKGRDDLRQDAVMQQVFQMCNTLLQRNTETRKRKLTICTYKV  1409
                           KKKKKKKKKKKKKKKKKKKKKKKKKKKKKKKKKKKKKKKKKKKKKK

TgATM        VPFSPAVGILEWVNDAVTLAQYLIGNPDNGYHGAHARYRPQDWTFTACRQKLRLAREKAL  1883
             VP S   G+LEW    V + ++L+ N D    GAH RYRP D++   C++K+   ++K+
HuATM        VPLSQRSGVLEWCTGTVPIGEFLVNNED----GAHKRYRPNDFSAFQCQKKMMEVQKKSF  1465
             KKKKKKKKKKKKKKKKKKKKKKKKKKKK    KKKKKKKKKKKKKKKKKKKKKKKKKKKK

TgATM        AQRRQMLSLGTADDGGLQKGQQKKEASGADAGSHSLVESLVQTYDEICDHFKPVLHHFFL  1943
              ++ ++                                     + ++C +F+PV  +F +
HuATM        EEKYEV-------------------------------------FMDVCQNFQPVFRYFCM  1488
             KKKKKK                                     KKKKKKKKKKKKKKKKK

TgATM        EHFPSADVWYEKRQRYRRTLAVSSMVSYIIGLGDRHTNNILLDVASGDLIHIDFGVVFEQ  2003
             E F    +W+EKR  Y R++A SS+V YI+GLGDRH  NIL++  S +L+HID GV FEQ
HuATM        EKFLDPAIWFEKRLAYTRSVATSSIVGYILGLGDRHVQNILINEQSAELVHIDLGVAFEQ  1548
             KKKKKKKKKKKKKKKKKKKKKKKKKKKKKKKKKKKKKKKKKKKKKKKKKKKKKKKKKKKK

TgATM        GKLLAIPELVPFRLTRDLVDGLGCLGVAGCFKRDCETTMEVLRRVSPLVVSIVEVLLFDP  2063
             GK+L  PE VPFRLTRD+VDG+G  GV G F+R CE TMEV+R     +++IVEVLL+DP
HuATM        GKILPTPETVPFRLTRDIVDGMGITGVEGVFRRCCEKTMEVMRNSQETLLTIVEVLLYDP  1608
             KKKKKKKKKKKKKKKKKKKKKKKKKKKKKKKKKKKKKKKKKKKKKKKKKKKKKKKKKKKK

TgATM        LYRWCLDPRRIL  2075
             L+ W ++P + L
HuATM        LFDWTMNPLKAL  1620
             KKKKKKKKKKKK
```

Fig. S1. **Sequence analysis of putative *T. gondii* ATM (TgATM) based on aminoacidic sequence. A**. Clustal W align of putative TgATM (TGME49_248530), Human ATM (AAB65827), TGME49_248530 ortholog of *Neospora caninum* (NCLIV_064490) and *Saccharaomyces cerevisiae* Tel1 (uniprot: P38110). F: FAT domain on human ATM; K: kinase domain in human ATM, and C: FATC domain on human ATM (Stracker et al, 2013). **B**. Blastp analysis of full length TGME49_248530 restricted to human taxid 9606. This putative TgATM retrieved truncated versions of several phosphatidylinositol 3-kinase homolog [Homo sapiens] sequences. The figure shows the alignment with Sequence ID: AAA86520.1. The expect score, identities and similarity (positives) is shown.

To note: TgATM shares an identity of 25% and positives of 43% in 506 bp, while putative TgATR shares an identity of 31% and positives of 48% with HuATR.
