# Supplementary figures and images for: Evaluation of ATM Kinase Inhibitor KU-55933 as Potential Anti-Toxoplasma gondii Agent
Source: Front Cell Infect Microbiol. 2019 Feb 13;9:26. doi: 10.3389/fcimb.2019.00026 (PMC6381018; doi:10.3389/fcimb.2019.00026)

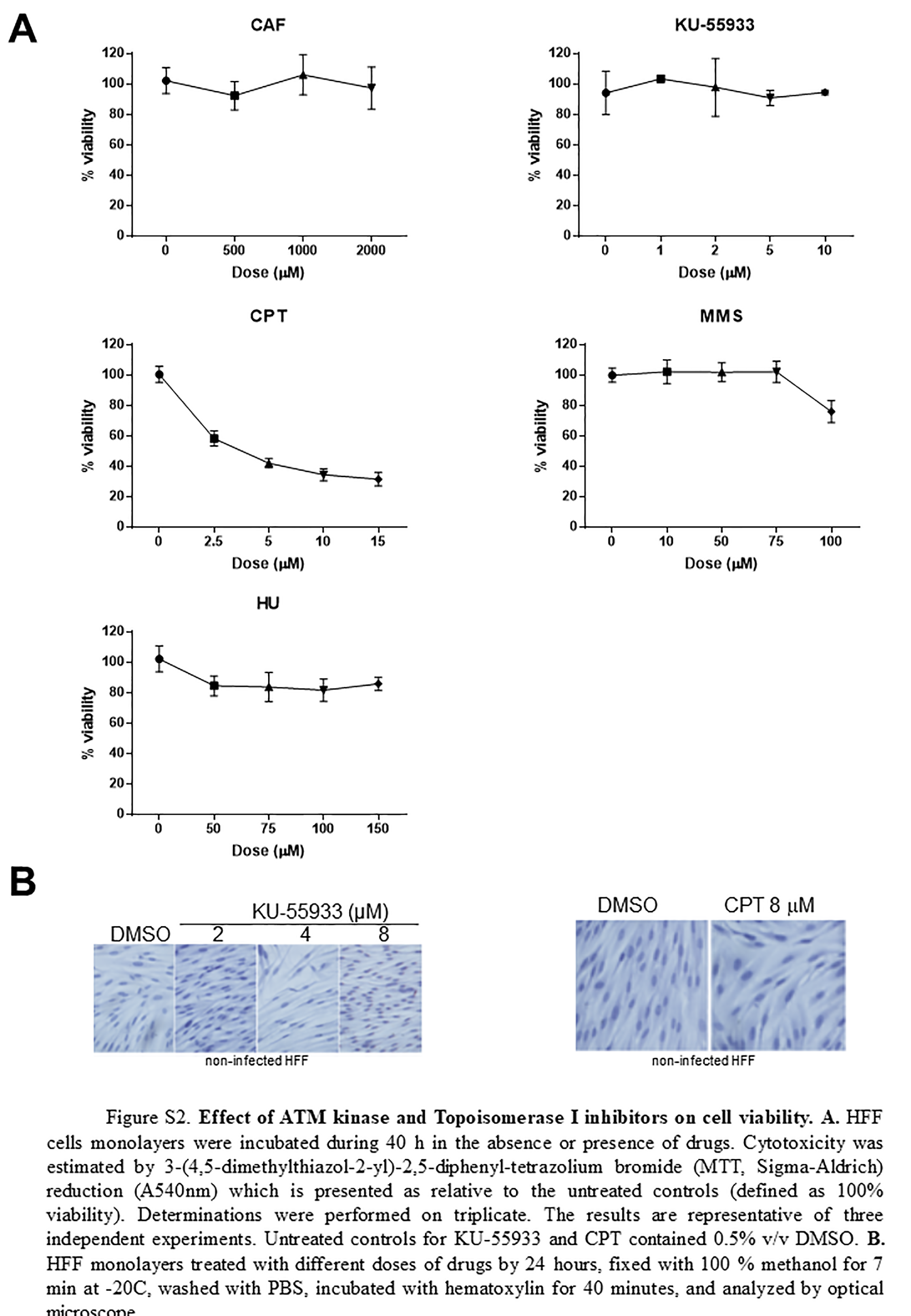

Supplement: Supplementary file 2 [file Image_2.tif]

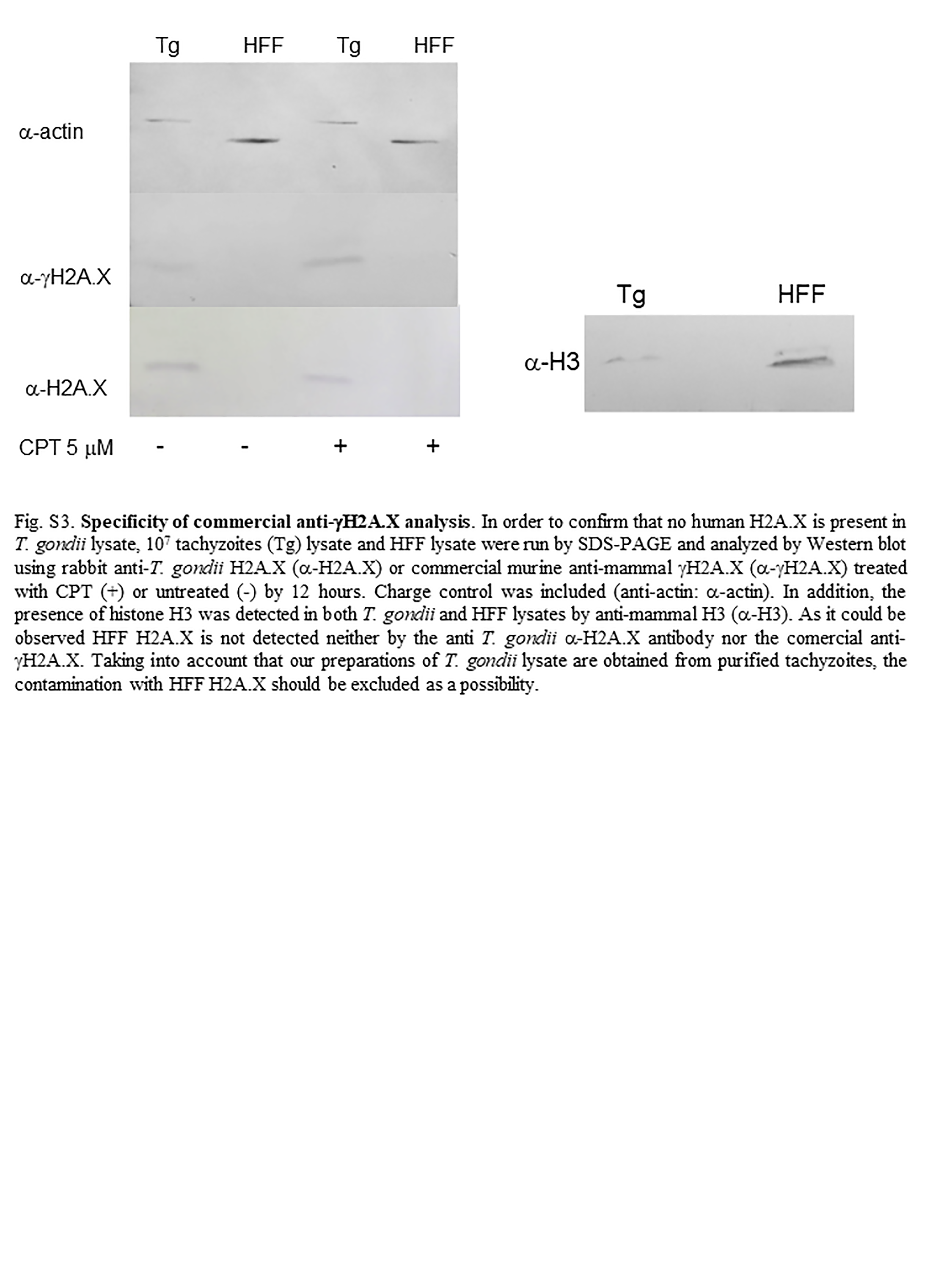

Supplement: Supplementary file 3 [file Image_3.tif]

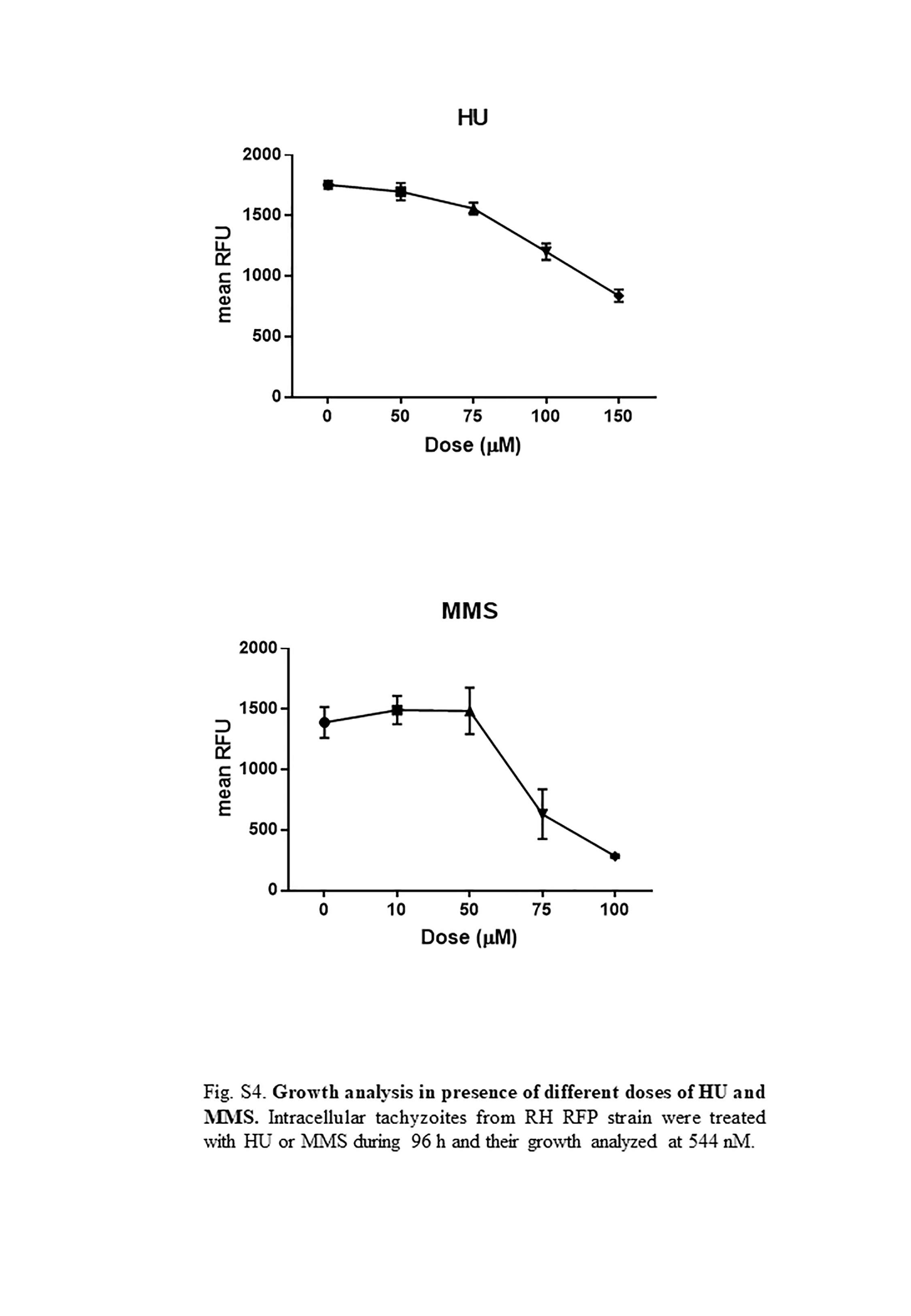

Supplement: Supplementary file 4 [file Image_4.tif]
